# Neural stem cells secrete factors facilitating brain regeneration upon constitutive Raf-Erk activation

**DOI:** 10.1038/srep32025

**Published:** 2016-08-24

**Authors:** Yong-Hee Rhee, Sang-Hoon Yi, Joo Yeon Kim, Mi-Yoon Chang, A-Young Jo, Jinyoung Kim, Chang-Hwan Park, Je-Yoel Cho, Young-Jin Choi, Woong Sun, Sang-Hun Lee

**Affiliations:** 1 Department of Biochemistry and Molecular Biology, College of Medicine, Hanyang University, Seoul, Korea; 2 Hanyang Biomedical Research Institute, Hanyang University, Seoul, Korea; 3 Department of Anatomy, College of Medicine, Korea University, Seoul, Korea; 4 Graduate School of Biomedical Science and Engineering, Hanyang University, Seoul, Korea; 5 Department of Biochemistry, BK21 PLUS Program for Creative Veterinary Science Research and Research Institute for Veterinary Science, College of Veterinary Medicine, Seoul National University, Seoul, South Korea; 6 ProtAnBio, Co., Seoul, South Korea; 7 Department of Microbiology, College of Medicine, Hanyang University, Seoul, Korea

## Abstract

The intracellular Raf-Erk signaling pathway is activated during neural stem cell (NSC) proliferation, and neuronal and astrocytic differentiation. A key question is how this signal can evoke multiple and even opposing NSC behaviors. We show here, using a constitutively active Raf (ca-Raf), that Raf-Erk activation in NSCs induces neuronal differentiation in a cell-autonomous manner. By contrast, it causes NSC proliferation and the formation of astrocytes in an extrinsic autocrine/paracrine manner. Thus, treatment of NSCs with medium (CM) conditioned in ca-Raf-transduced NSCs (Raf-CM; RCM) became activated to form proliferating astrocytes resembling radial glial cells (RGCs) or adult-type NSCs. Infusion of Raf-CM into injured mouse brains caused expansion of the NSC population in the subventricular zone, followed by the formation of new neurons that migrated to the damaged site. Our study shows an example how molecular mechanisms dissecting NSC behaviors can be utilized to develop regenerative therapies in brain disorders.

Neural stem cells (NSCs) that arise from a single neuroepithelial cell layer of the neural tube initially multiply for self-renewal during early brain development. After an appropriate number of NSCs have formed, neuronal differentiation of the NSCs commences and is followed by differentiation to astroglial cell types at a later time during embryonic development (for review, see ref. 1). Cells characterized by glial marker expression and long radial processes (called radial glial cells; RGCs) appear during the neurogenic period of brain development. These cells undergo asymmetric cell divisions into neurons and NSCs in the ventricular zone (VZ), and the newly formed neurons migrate along the radial processes towards the surface of the brain. Thus, the RGCs serve as guides for neuronal migration and as neurogenic NSCs. After development is complete, a portion of NSCs remains in several regions of the adult mammalian brain such as the subventricular zone (SVZ) of the lateral ventricles, the hippocampal dentate gyrus, and the subcallosal white matter, and new neurons are formed in these adult brain regions (for review, see ref. 2). The adult NSCs, like embryonic RGCs, have astroglial phenotypes. There is compelling evidence that the adult NSCs originate from embryonic RGCs^3^ and embryonic NSCs^4^,^5^. In response to brain damage, adult NSCs present in the SVZ (SVZ-NSCs) take part in the regenerative processes by multiplying and undergoing neuronal differentiation, along with migration towards the lesion sites (for review, see ref. 6).

A number of cytokines/growth factors have been shown to modulate NSC behaviors in the developing and adult brain. Of these, fibroblast growth factors (FGFs), cytokines and their receptors, which are widely expressed in the developing and adult brains (for review, see ref. 7), have been extensively studied. When FGF binds to its receptor, the activated receptor tyrosine kinase (RTK) triggers an intracellular phosphorylation cascade that involves the signaling molecules Ras, Raf, and Erk and ultimately controls various cellular events. FGFs promote NSC behaviors ranging from NSC proliferation^8^,^9^ to neuronal^10^,^11^ and astroglial differentiation^12^,^13^,^14^ by activating intracellular Ras-Raf-Erk signaling. Many other factors also promote NSC proliferation and differentiation by activating Raf-Erk signaling^1^. NSC proliferation is inhibited by differentiation stimuli^15^,^16^,^17^. In addition, neuronal vs. astrocytic differentiation occurs at the expense of the other during brain development^18^,^19^. Thus, proliferation vs. differentiation and neuronal vs. astrocytic differentiation are regarded as opposing NSC behaviors. How Raf-Erk signaling triggers the multiple and opposing NSC behaviors is not known.

The aim of this study was to address this issue and ultimately obtain clues as to how to differentially manipulate NSC behavior. We show here that Raf-Erk activation in NSCs intrinsically promotes neuron differentiation, whereas it causes NSC proliferation and astrocytic differentiation in a paracrine/autocrine manner. Thus, factors released from NSCs upon Raf-Erk activation induce the formation of proliferating RGC-like astrocytes, which can participate in the brain regeneration process. The information obtained not only furthers our understanding of brain development but also aids in regenerative medicine.

## Results

### Cell proliferation/anti-neuronal differentiation induced by Raf-Erk activation at high NSC densities

Previous studies have identified the role of FGF-Raf-Erk signaling in NSC behaviors by treating NSCs with FGF1 or 2^20^,^21^,^22^,^23^. FGF not only activates Raf-Erk signaling but also other major intracellular signaling components such as PI3K-Akt, PLC_γ_, Jak-STAT, and IKK-NFkB (for review, see refs 24,25). Such complexity makes it difficult to identify the individual contributions to the observed findings. To avoid this, we activated the Raf-Erk intracellular pathway at a downstream level by over-expressing a constitutively active form of Raf (ca-Raf)^26^. We transduced NSC cultures derived from the cortices of rat embryos at embryonic day 14 (E14) with retroviruses expressing ca-Raf, and examined their proliferation and neuronal and astrocytic differentiation under different culture conditions and in response to different treatments.

**Figure 1:**
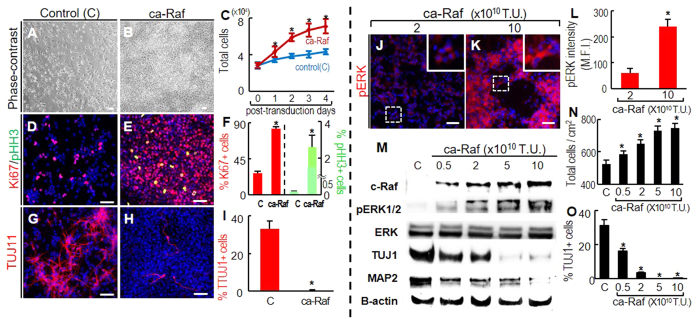
Raf-Erk signal activation at high NSC densities induces cell proliferation and inhibition of neuronal differentiation. (**A–I**), To obtain cultures at 50–80% cell confluence, NSCs from E14 rat embryonic cortices were plated at 3–4 × 10^4^/cm^2^ in FGF2-supplemented N2 medium, and transduced with ca-Raf or control virus (viral titers: 10 × 10^10^ transduction unit, TU) on the following day. The cells were further cultured in N2 (without FGF2) for 4 days. Cell numbers on post-transduction days 0–4 (**A–C**), and numbers of cells positive for the proliferation markers Ki67 and M-phase pHH3 (**D–F**), and for the neuronal marker TUJ1 (**G–I**) were counted on post-transduction day 4. *p < 0.001, n = 3, Student’s t-test. Scale bar = 50 μm. (**J–O**), Dose-dependent ca-Raf effects on proliferation and neuronal differentiation. Levels of Erk activation were readily controlled by varying ca-Raf viral titers (**J–M**). Cortical NSC cultures (plated at 4 × 10^4^/cm^2^) were transduced with 0.5–10 × 10^10^ TU of ca-Raf virus, and stained with fluorescent pERK1/2 antibody 2 days later (**J–L**). Insets, high-magnification images of the boxed areas. Scale bar = 50 μm. Erk activation in individual cells was estimated from the mean fluorescence intensity (MFI) of individual pERK1/2 -stained cells using LAS image analysis (Leica). 54(2 × 10^10^ TU) and 63(10 × 10^10^ TU) stained cells were selected at random, and the margins of the individual cells were drawn. MFIs within the outlined area were calculated by comparison with the overall background intensity (**L**). *p < 0.001, Student’s t-test. Erk activation (pERK1/2 protein level) in cultures transduced with different titers of ca-Raf virus was further analyzed by western blotting (**M**). Dose-dependent Raf-Erk effects on cell proliferation and neuronal differentiation were estimated by counting total viable cells (**N**) and neurons positive for TUJ1 (**O**) in cultures 6 days after transduction. *p < 0.001, n = 3 cultures, one-way ANOVA.

Embryonic cortical NSC cultures with confluent cell densities were transduced with ca-Raf, and cell proliferation/differentiation was examined in N2 medium over the following 4 days. The transduced cells multiplied more rapidly than mock-transduced control cultures (Fig. 1A–C), and contained a higher percentage of cells positive for Ki67 (proliferation-specific) and pHH3 (M-phase-specific) on day 4 (Fig. 1D–F). The ca-Raf-transduced cultures produced fewer TUJ1+ neurons than the control cultures (Fig. 1G–I). These findings are consistent with the general concept that NSC proliferation/stem cell maintenance is induced by Ras-Raf-Erk activation upon FGF2 treatment (10–20 ng/ml) (for review, see ref. 27).

In contrast, another line of study revealed the opposite effect of FGF2 on cultured NSCs, namely enhanced neuronal differentiation^28^,^29^. Because neuronal differentiation in previous studies was observed at lower concentrations of FGF2 (0.1–1 ng/ml), we examined if different levels of Raf-Erk signaling had opposite effects. Levels of Raf-Erk signal activation in individual (Fig. 1J–L) and total cells (Fig. 1M), estimated by phosphorylated ERK1/2 (pERK1/2) levels in immunocytochemical and western-blot analyses, respectively, were readily controlled by the titer of the ca-Raf viruses used for NSC transduction. The proliferative/anti-neurogenic effects became less prominent in response to lower ca-Raf titers, but never resulted in increased neuronal differentiation (Fig. 1M–O); protein levels of the neuronal markers TUJ1 and MAP2 (Fig. 1M) and % TUJ1+ neurons (Fig. 1O) continued to be lower in cultures transduced with even the lowest ca-Raf titers than in the mock-transduced controls.

### Activated Raf-Erk signaling induces neuronal differentiation of NSCs at low cell densities

**Figure 2:**
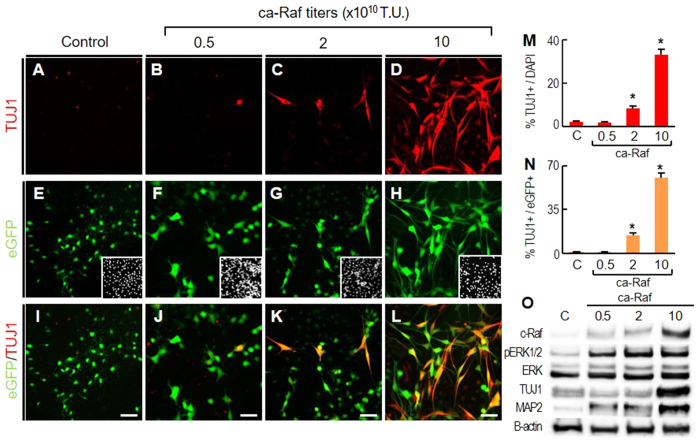
Raf-Erk signaling in NSCs induces neuronal differentiation in a cell-autonomous manner. NSCs derived from rat embryonic cortices were cultured at low cell density (5–10% cell confluence). These low density cultures were transduced with bicistronic *ca-Raf-IRES-eGFP* (0.5, 2, and 10 × 10^10^TU) or control (eGFP, **A,E,I**). After four days, %TUJ1+ among DAPI+ cells (**M**) and % TUJ1+ among eGFP+ cells (**N**) were estimated. *p < 0.001, n = 4, one-way ANOVA. Insets of (**E–H**), DAPI+ cell images of the same microscopic fields. Erk activation and TUJ1 and MAP2 protein were also measured by WB (**O**).

Cell density is one of the critical factors affecting NSC behavior^30^,^31^,^32^. The extrinsic effects of cell-cell contact and auto/paracrine factors in confluent cell cultures are diminished by reducing cell density. Strikingly, when NSCs derived from embryonic cortices were cultured at low cell densities, the effect of Raf-Erk activation on neuronal differentiation was the opposite of that seen at high cell densities: TUJ1+ neuronal numbers on day 4 in the cultures transduced with ca-Raf far exceeded those in the control, and the neuronal yield was further increased at higher titers of ca-Raf transduction (for instance, %TUJ1+ neurons among total DAPI+ cells: 33.4 ± 2.4% in ca-Raf-transduced vs 2.1 ± 0.7% in control-transduced cultures at 10 × 10^10^ transduction unit (TU), Fig. 2A–D,M). The ca-Raf dose-dependent increase in neuronal yields at low cell density was confirmed by western-blot analyses for the neuron-specific proteins TUJ1 and MAP2 (Fig. 2O). When the transduced cells were labeled with enhanced green fluorescent protein (eGFP) using the bicistronic vector carrying *ca-Raf-IRES-eGFP* (Fig. 2E–L), virtually all the TUJ1+ cells in the ca-Raf-transduced cultures were positive for eGFP (Fig. 2I–L) and % TUJ1+ cells among the transduced eGFP+ cells increased at the higher viral titers (Fig. 2N). Portions of the ca-Raf-transduced eGFP+ cells expressed the mature neuronal maker MAP2 and NeuN after longer period of differentiation, but not in the cultures transduced with the control eGFP vector (Fig. S1). Our interpretation of these results is that Raf-Erk activation in NSCs promotes their neuronal differentiation in a cell-autonomous manner; however this intrinsic neurogenic effect is overridden by the extrinsic anti-neurogenic effects activated in the cultures at high cell densities. We have further found that activated CREB acts in concert with other molecules downstream of Raf-Erk in neuronal differentiation (Fig. S2). The intrinsic neurogenic effects of Raf-Erk activation are described further in the discussion section.

### Activation of Raf-Erk in embryonic NSCs induces cell proliferation as well as astroglial differentiation in an autocrine/paracrine manner

**Figure 3:**
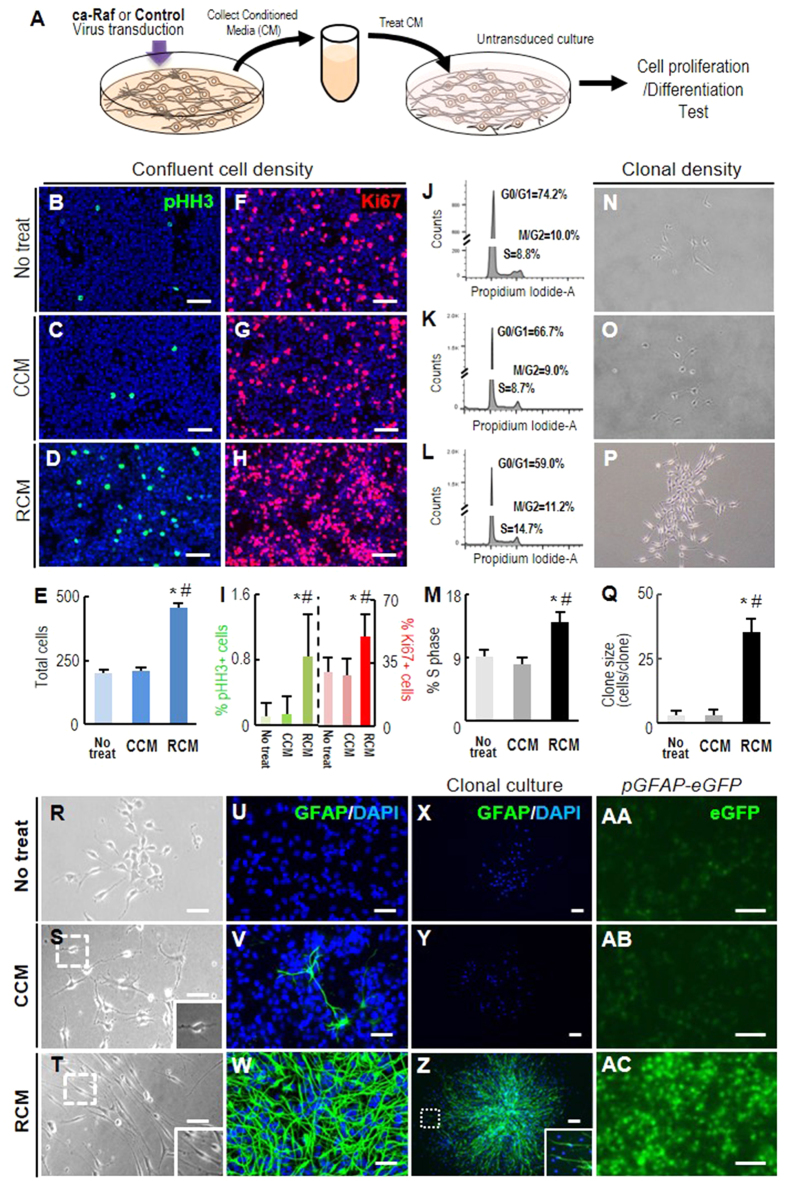
Cortical NSCs transduced with ca-Raf secrete factors that promote cell proliferation and astrocytic maker expression. (**A**) Schematic of the experimental procedure. NSCs derived from rat embryonic cortices were cultured at high cell density (80–90% cell confluence) and transduced with ca-Raf or control viruses (10 × 10^10^TU). Media conditioned by the transduced cultures were collected and non-transduced NSC cultures were exposed to the collected media as described in ‘Materials and Methods’. (**B–Q**) Cell proliferation estimated from total cell numbers (**B–E**), %pHH3+ cells (**B–D,I**), %Ki67+ cells (**F–H,I**), and % of cells present in S-phase measured by FACS analysis (**J–M**) grown at normal confluent cell density (50–70% cell confluence). Cell proliferation was further estimated by sizes of clones (cells/clone) formed 4 days after plating NSCs at a clonal density (1000 cells/6-cm dish) (**N–Q**). Significant different from the untreated* and CCM-treated# at *p* < 0.001, n = 3 cultures, one-way ANOVA. R-AC, Cell morphology (**R–T**) and GFAP expression (U-AC). Expression of the astroglial marker GFAP was assessed with an antibody against GFAP in the cultures plated at normal (**U–W**) and clonal density (**X–Z**) and by eGFP-filtered microscopic examination in cultures derived from *pGfap-eGFP* mouse embryonic cortices (AA-AC). Insets, enlarged images of the boxed areas. Scale bars, 100 μm.

The ca-Raf-mediated stimulation of cell growth, observed in the embryonic NSC cultures at high cell densities, gradually declined as cell density decreased, so that at the lowest plating cell density tested (2 × 10^2^ cells/cm^2^) total cell numbers in the ca-Raf-transduced cultures were not significantly different from those in the controls (Fig. S3). Protein concentrations (0.15 ± 0.03 mg/ml) were greater in the media collected from the ca-Raf-transduced cultures plated at higher cell density (4 × 10^4^ cells/cm^2^) than those (0.04 ± 0.006 mg/ml) at lower cell density (1 × 10^4^ cells/cm^2^), indicating that the Raf-Erk effect on cell proliferation is likely mediated by secreted proteins. In order to test the possibility of paracrine/autocrine signaling, we prepared medium conditioned in confluent NSC cultures transduced with ca-Raf or with control virus, and incubated NSCs in the conditioned media (CM, 0.1–0.13 mg of protein/ml) (Fig. 3A). Incubation in the CM conditioned by ca-Raf-transduced cells (Raf-CM; RCM), but not in the control CM (control-CM; CCM), greatly stimulated cell proliferation as shown by total cell numbers (Fig. 3B–E) and % cells positive for Ki67 (Fig. 3F–I) and for pHH3 (Fig. 3B–D,I) after 4 days in CM. Consistent with this, FACS analysis showed that the proportion of cells in S phase was higher in the RCM-treated culture (Fig. 3J–M). Moreover when cells were plated to form individual clones, the clone sizes (cells/clone) were much greater in the presence of RCM than in the presence of CCM (35.19 ± 6.1 cells/clone vs 3.12  ± 2.5 cells/clone, n = 40 clones counted in each group, Fig. 3N–Q).

Most importantly, after 2–3 days in RCM, the NSCs underwent a change in shape into elongated cells with a radial morphology (Fig. 3T) and virtually all of those cells expressed the astroglial marker glial fibrillary acidic protein (GFAP)(Fig. 3W,Z). Similarly RCM induced eGFP expression in NSC cultures derived from the cortices of mouse embryos in which eGFP expression was under the control of the *Gfap* promoter (*pGfap-eGFP*) (Fig. 3AA-AC). These findings indicate that intracellular Raf-Erk signaling in NSCs causes them to secrete diffusible auto/paracrine factors promoting not only their proliferation, but also the expression of astrocytic markers.

### RCM-treated GFAP+ cells have RGC/adult-NSC-like properties

**Figure 4:**
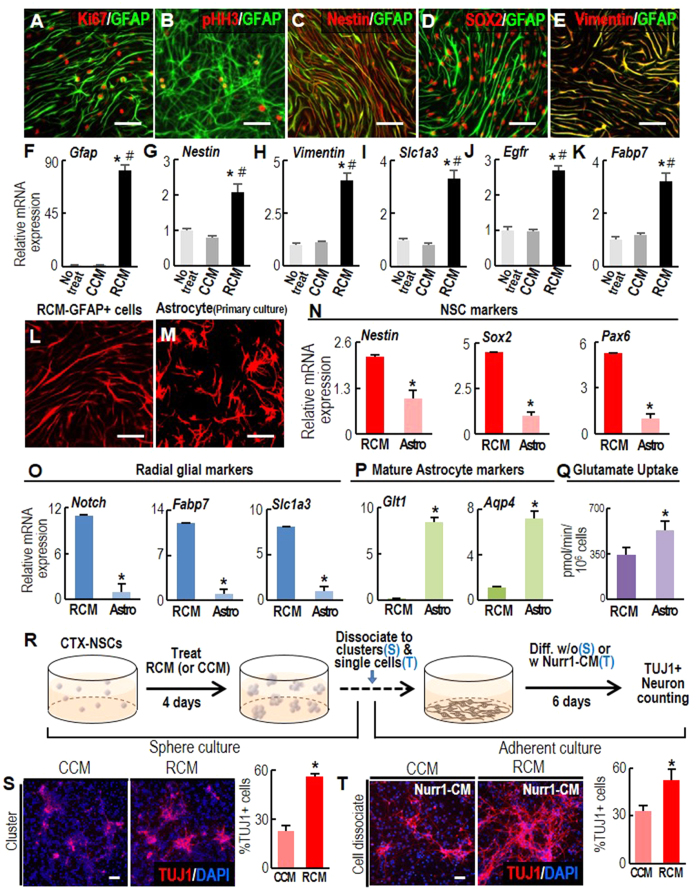
Radial glial- or adult NSC-like properties of the GFAP+ cells generated by Raf-CM treatment. (**A–K**) NSC marker expression in the GFAP+ cells generated by RCM treatment (RCM-GFAP+ cells). Immunocytochemical (**A–E**) and real-time PCR (**F–K**) analyses were used to compare marker expression in RCM- and CCM-treated cultures. Significant different from the untreated* and CCM-treated# at p < 0.005, n = 3, one-way ANOVA. (**L–Q**), Morphology (**L,M**), marker expressions (**N–P**), and glutamate uptake activity (**Q**) in RCM-GFAP+ cells (RCM) and primary-cultured astrocytes (Astro). Differentiated astrocytes were cultured from mouse cortex on postnatal day 5. *p < 0.005, n = 3–5, Student’s t-test. (**R–V**), Neurogenic potential of RCM-GFAP+ cells. (**R**), Schematic of the experimental procedure. RCM- and CCM-treated neurospheres were dissociated into small clusters (**S**) or single cell dissociates (**T**), and plated at identical cell densities in N2 media. Neuronal differentiation was examined without (**S**) or with Nurr1-CM treatment (**T**). After six days, neuron yields were assessed as % TUJ1+ cells among total DAPI+ cells. *p < 0.001. n = 3–6 cultures, Student’s t-test. Scale bars, 50 μm.

The combined effects of RCM treatment on cell proliferation and astrocyte marker expression generated proliferating GFAP+ cells expressing proliferating cell markers (Fig. 4A,B). In addition, it is noted that markers specific for adult NSCs and RGCs, such as Nestin, SOX2, and Vimentin (Fig. 4C–E), co-localized in the GFAP+ cells generated by RCM treatment (RCM-GFAP+ cells) and there was increased expression of transcripts of these markers (Fig. 4F–K). Moreover, the elongated and radial shape of the RCM-GFAP+ cells (Figs 3T,W,Z and 4L) is a morphological feature of RGCs^33^. Their shape was clearly distinct from the typical stellate and flat morphology of the prototypic astrocytes primarily derived from the brain of mouse pups (Fig. 4M). The expression of RGC-specific markers (*Nestin*, *Sox2*, *Pax6*, *Notch*, *Fabp7*, *Slc1a3*) was much higher in the RCM-GFAP+ cells than in primary astrocytes, whereas that of markers specific for mature astrocytes [*GLT1*, Aquaporin 4 (*Aqp4*)] was lower (Fig. 4N–P). Glutamate transport activity, which is a functional characteristic of mature astrocytes, was significantly higher in the primary astrocyte cultures than in the RCM-GFAP+ cells (Fig. 4Q). These findings collectively suggest that RCM-induced GFAP+ astrocytes resemble NSCs rather than mature astrocytes.

### The neurogenic potential of the RCM-GFAP+ cells

A characteristic distinguishing RGCs and adult-type NSCs from prototypic astrocytes is their neurogenic potential. They are quiescent, but give rise to neurons in response to neurogenic stimuli such as brain injury (adult NSCs) and developmental cues (RGCs) (for reviews, see ref. 34,35). To test for neuron formation from the RCM-GFAP+ cells, cells treated with RCM (or CCM as control) were fed fresh N2 medium and allowed to differentiate in the absence of CM. Cell density is an important factor affecting neuronal differentiation but, as we have seen, increases more after RCM treatment than after CCM treatment (Fig. 3E). Therefore, to achieve similar cell densities in the CCM- and RCM-treated cultures during the differentiation period, the NSCs derived from embryonic cortices were cultured as floating cell aggregates (neurospheres) in the presence of RCM or CCM. After 4 days the spheres were dissociated into small clusters (average size: 50–150 μm) or isolated single cells, plated in FN-coated culture dishes at the same densities, and left to differentiate for 6 days in N2 medium (Fig. 4R). In these culture conditions, cell density during differentiation was not significantly different between the cultures. In the cultures plated with small clusters, greater neuronal differentiation was evident in the RCM-treated cell clusters, as shown by the higher %TUJ1+ neurons (Fig. 4S). Consistent with previous studies^36^, only a few neurons formed from the single dissociated cells, suggesting that NSC differentiation requires adequate cell density and cell-cell contact. In this non-neurogenic condition, numbers of TUJ1+ neurons were only slightly greater in the dissociated RCM-pretreated cells than in the dissociated CCM-treated cells (data not shown). It appeared that this finding could reflect some quiescent characteristic of RGCs and adult NSCs per se, and that they might require extrinsic or intrinsic neurogenic cues in order to form neurons. We therefore asked if we could provoke the dissociated RCM-GFAP+ cells to form neurons by means of extrinsic neurogenic stimuli. We have shown previously that the nuclear hormone receptor Nurr1 (NR4A2) in NSCs induces the synthesis and secretion of paracrine factors promoting neuronal differentiation^37^. Thus, we examined the effect of CM prepared from Nurr1-expressing NSCs (Nurr1-CM) on dissociated RCM-GFAP+ cells. Treatment of the dissociated cells with Nurr1-CM during differentiation increased neuronal yields to a significantly greater extent in the cultures pre-treated with RCM compared with that in the CCM-treated cultures (Fig. 4T). Together, these findings demonstrate that the RCM-GFAP+ cells possess neurogenic potential under neurogenic conditions.

### RCM treatment promotes neurogenesis from GFAP+ NSCs in the SVZ of adult mice

We next examined the effect of RCM on NSCs (GFAP+) derived from adult brain. To this end, NSCs were isolated from the SVZ of the adult mouse lateral ventricle (LV), and cultured in a form of floating neurospheres (Fig. S4A). As in the case of embryonic NSC cultures, RCM treatment enhanced GFAP+ cells in the primary neurospheres (Fig. S4B–E). In neurosphere forming assays (Fig. S4F), size and number of neurospheres (secondary and tertiary) were greater in RCM-treated cultures, compared to those of CCM-treated (Fig. S4G–K), indicating a RCM-mediated increase in cell proliferation and prevalence of proliferating NSCs. When the neurospheres were induced to differentiate by plating them on FN-coated dishes, significantly more TUJ1+ neurons were formed from the RCM-treated adult neurospheres than from the CCM-treated ones (Fig. S4L).

**Figure 5:**
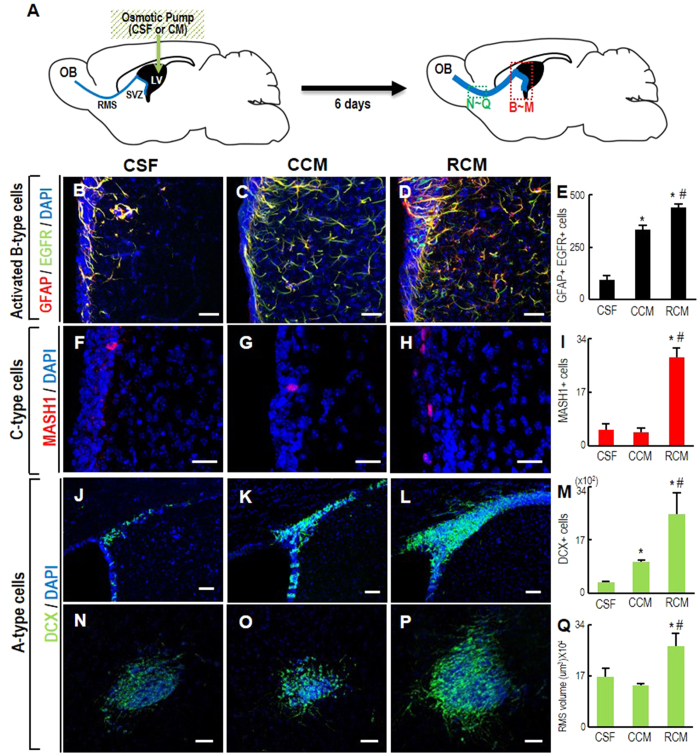
RCM treatment enhances adult neurogenesis. (**A**) Schematic presentation of the experimental procedure. RCM-effects on SVZ-NSC proliferation and neurogenesis were assessed in brains of adult mice. CM was infused into the right lateral ventricle (LV) using an osmotic pump. Six days after CM infusion, activated B-type (**B–E**), C-type (**F–I**), and A type cells (neuroblasts) (**J–M**) in the ipsilateral SVZs and A-type cells of the RMS (N-Q) were assessed. Brain regions analyzed are indicated with dotted boxes in the right schematic of ‘(**A**)’. Cerebrospinal fluid (CSF) was infused into the control mice. Significantly different from the CSF-infused control* and from CCM-infused # at *p* < 0.005, one-way ANOVA, n = 4 (CSF), 7 (CCM), and 7 animals (RCM).

To examine the effect of RCM on *in vivo* neurogenesis in the adult brain, we infused RCM into the right LV of adult mouse brains and analyzed the SVZ and rostral migratory stream (RMS) (Fig. 5A), in which newly formed neurons from the SVZ migrate towards the olfactory bulb. During neurogenesis of the SVZ-NSCs, type B NSCs are activated (GFAP+,EGFR+), and sequentially transform first into C cells (MASH1+), and then into A cells (neuroblast/newly formed migrating neurons, doublecortin+ (DCX+)). Six days after RCM infusion, the numbers of activated B cells and C cells in the SVZ were much higher than in the SVZ of control mice infused with cerebrospinal fluid (CSF) or CCM (Fig. 5B–I). Accordingly the number of DCX+ neuroblasts was also greater in the SVZ of the RCM-infused brains (Fig. 5J–M), along with an increase in the DCX+RMS volume (Fig. 5N–Q). CCM infusion also led to an increase in GFAP+, EGFR+ activated B cells and DCX+ A cells in the SVZ, but to a smaller extent than those in the RCM-treated cultures. Furthermore, there was no significant change in numbers of C-type cells and migrating DCX+ neuroblasts, indicating that it is unclear whether CCM induces neurogenesis in SVZ-NSCs. The RCM-promoted proliferation and neurogenesis were further confirmed in the neurosphere cultures derived from the CM-infused SVZs (Fig. S5A). In sphere-forming assays, cell dissociates derived from RCM-infused SVZs yielded more and larger spheres than those derived from CCM-infused SVZs (Fig. S5B–E). In addition, when the spheres were attached and induced to differentiate, the number of the spheres containing TUJ1+ neurons (TUJ1+ clones) was greater in the cultures derived from the RCM-infused SVZs (Fig. S5F).

**Figure 6:**
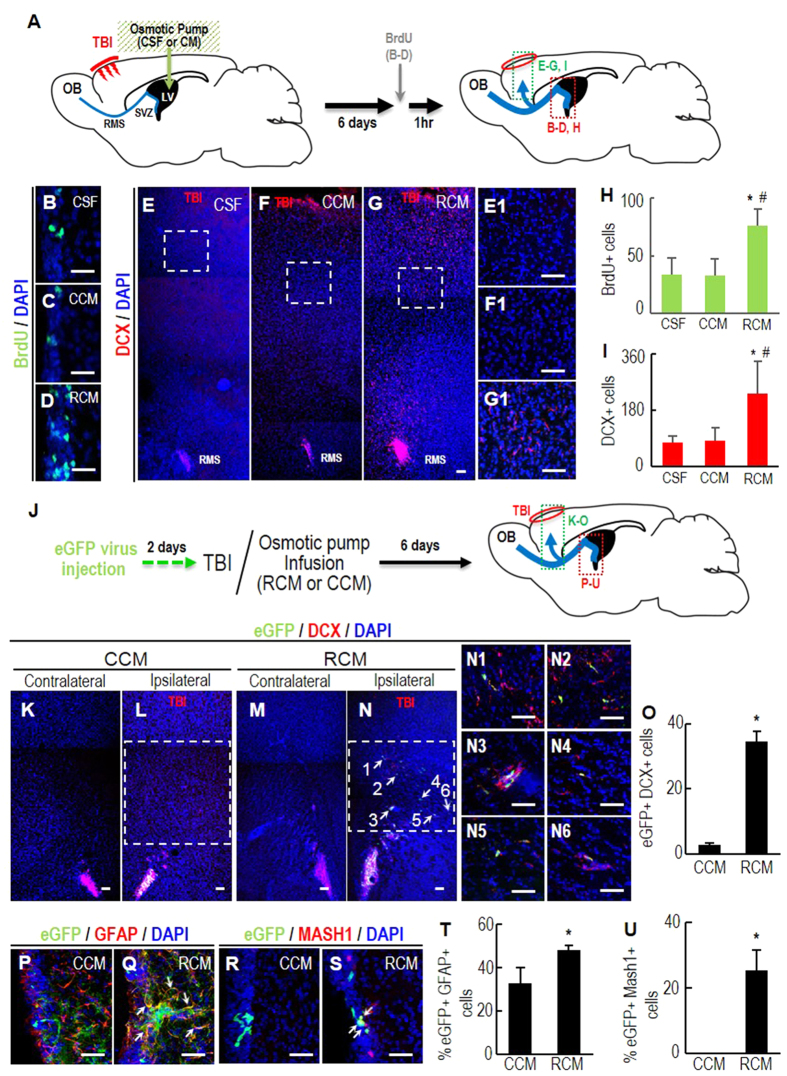
Enhanced SVZ-NSC neurogenesis and neuronal migration to the damaged brains by RCM treatment. (**A**) Schematic presentation of the experimental procedure in (**B–I**). Cryogenic TBI was applied to the right cortical brain and followed by infusion of CM (or CSF) into ipsilateral LV. All analyses were carried out 6 days after TBI. (**B–D,H**) SVZ-cell proliferation determined by the numbers of cells immunoreactive for BrdU 1 hr after BrdU injection. (**E–G,I**) Numbers of DCX+ neuroblasts migrating to the TBI region. (**E1–G1**) High-powered images of the boxed areas in E-G. Significantly different from the CSF(*)- and CCM (#)-infused animals at *p* < 0.001. n = 5 (CSF), 7 (CCM), and 7 (RCM) mice. Scale bars, 50 μm. (**J–U**) SVZ-NSC origin of the migrating neuroblasts confirmed by labeling SVZ-NSCs with eGFP. (**J**) Schematic presentation of the experimental procedure in (**K–U**). Two days prior to the TBI (and CM infusion), eGFP-expressing retroviruses were injected to the right SVZ. Six days after TBI, eGFP+/DCX+ cells in the migrating areas (in green box, (**K–O**) and (**B,C**) type cells in the ipsilateral SVZs (in red box, **P–U**) were counted. Shown in (**K–N**) are representative ipsilateral and contralateral (negative control) brain sections of RCM- and CCM-infused mice. (**N1–N6**) High-powered views of eGFP+/DCX+ cells indicated by arrows in (**N**). Shown in (**P–U**) are representative images of eGFP/GFAP, eGFP/MASH1-double positive (**B,C**) cells in the ipsilateral SVZ. Double positive cells in (**Q,S**) are indicated by arrows. Scale bar, 50 μm. *p < 0.001, n = 5 mice for each group.

It has been reported that, in response to brain injury, NSCs in the adult SVZ proliferate and differentiate into neuroblasts that migrate to the injury site. When cryogenic traumatic brain injury (TBI) was applied to the cortical brain followed by infusion of CM into the right LV (Fig. 6A), we found >2 fold more 5′-bromo-2-deoxyuridine (BrdU)-labeled NSCs in the SVZ of the mouse brains 6 days after RCM infusion than CSF- or CCM-infused controls (Fig. 6B–D,H). In addition, a greater number of DCX+ neuroblasts appeared in the regions approaching the TBI site in the brains infused with RCM (Fig. 6E–G,I). DCX+ neuroblasts can be derived by local activation of astrocytes in the injury site^38^. To rule out the possibility, the SVZ-NSCs were labeled with eGFP by injecting retroviruses expressing eGFP 2 days prior to the TBI (Fig. 6J). Along with an increase of eGFP/GFAP, eGFP/MASH1-double positive B and C cell populations in the ipsilateral SVZ (Fig. 6P–U), the number of eGFP/DCX-double positive cells at the injury sites 6 days after TBI was greater in the animals infused with RCM that in those receiving CCM (Fig. 6K–O), thus confirming the effect of RCM on SVZ-NSC neurogenesis after injury. Taken together, these results suggest that RCM treatment promotes neurogenesis in the damaged brain and could be used in strategies for brain repair.

### Molecules responsible for the induction of proliferating astrocytes by RCM

**Figure 7:**
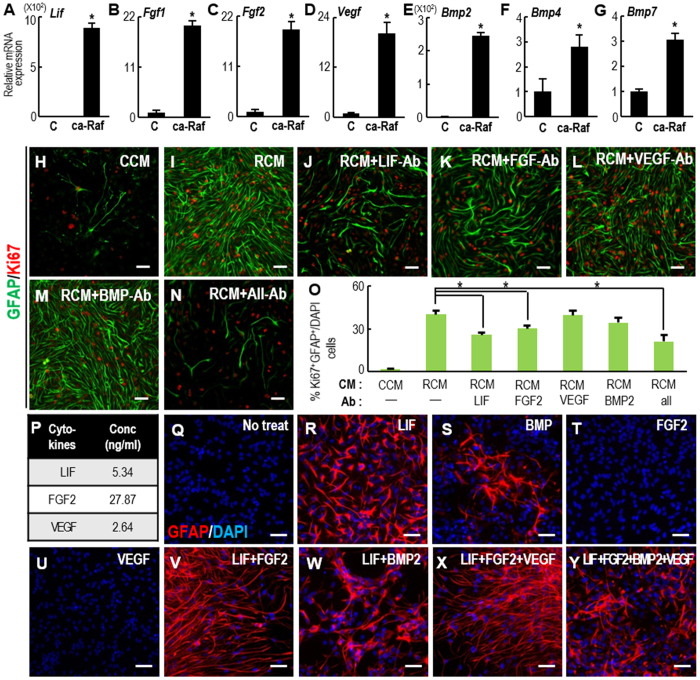
Molecules responsible for RCM induction of proliferating astrocytes. (**A–G**) Real-time PCR analyses of candidate molecules known to induce NSC proliferation and astrocytic differentiation. mRNA expression was compared between NSCs transduced with the control and ca-Raf virus. *Significance at *p* < 0.01, n = 3–6 reactions, Student t-test. (**H–O**) Treatment with blocking antibody to test its effect on RCM induction of proliferating astrocytes (GFAP+, Ki67+ cells). E14 cortical NSCs were treated with CCM (**H**) or RCM in the absence (**I**) or presence (**J–N**) of the blocking antibodies for 4 days. The antibodies used were anti- LIF, FGF2, VEGF, and BMP (0.1 mg/ml for all above ab). *Significantly different from RCM-treated cultures at *p* < 0.01, one-way ANOVA, n = 3–5 cultures. (**P**) Cytokines in the RCM determined with a Bio-plex^®^ 200 System. (**Q–Y**) Morphology of GFAP+ cells induced by treatment with the indicated cytokines. Cortical NSC cultures were treated with the concentrations of LIF, FGF2, and VEGF determined in (**P**), and 10 ng/ml of BMP2. Scale bars, 50 μm.

To gain insight into which molecules are responsible for the RCM effects, we examined the expression of secretory molecules that have been reported to induce NSC proliferation and astrocytic differentiation. We found that transcripts of the soluble factors leukemia inhibitory factor (LIF), FGF1, 2, vascular endothelial growth factor (VEGF), and bone morphogenetic protein (BMP) 2, 4, and 7 were dramatically higher in ca-Raf-transduced NSCs than in control NSCs (Fig. 7A–G). Among these molecules, we focused on LIF expression, because in addition to the known roles of LIF in astrocytic differentiation/proliferation of NSCs and in brain repair^39^,^40^, a previous study had obtained findings similar to ours demonstrating LIF expression/secretion upon ca-Raf transduction and a crucial role of LIF in thyroid cancer cells^41^. LIF treatment activates the Jak-STAT pathway, and homodimers of activated (phosphorylated) STAT (pSTAT) are recruited to the STAT binding site (TTCCGAGAA) of the *Gfap* promoter^42^. The number of GFAP+/Ki67+ proliferating astrocytes in RCM-treated cultures was greatly reduced by treatment with LIF-blocking antibody (Fig. 7I,J,O). Furthermore, in examining the role of STAT in RCM-mediated effects, we found that the RCM effect on GFAP+ cell yields was drastically reduced in NSCs overexpressing a dominant negative (dn)-STAT3 (Fig. S6A–D). RCM treatment strongly increased luciferase activity driven by the *Gfap* promoter in NSC cultures, but the RCM effect was greatly reduced when the STAT binding site in the *Gfap* promoter was deleted (*pGfap*-*STAT mutant*) (Fig. S6E,F). These findings collectively show that upon intracellular activation of Raf-Erk, NSCs secrete LIF, which in turn can induce the generation of proliferating astrocytes from neighboring NSCs via the Jak-STAT pathway. In agreement with this, a recent study has shown that intracellular Ras-Raf-Erk activation using an optogenetic tool stimulates the secretion of ligands that activate Jak-STAT3 signaling in neighboring cells^43^.

We also observed a reduction in proliferating astrocyte numbers in cultures treated with antibodies against FGF, VEGF, and BMP, although the change was signficant only with the FGF antibody (Fig. 7H–O). We measured the amounts of LIF, FGF2, and VEGF proteins using a Luminex system (Bio-plex^®^200 Systems) (Fig. 7P) and used the corresponding concentrations in the following *in vitro* and *in vivo* experiments. As BMP protein levels could not be measured by the Luminex system, we used 10 ng/ml of BMP2, a concentration that has been reported to induce astrocytic differentiation^44^. LIF and BMP2 efficiently induced GFAP+ cells (Fig. 7R,S). However, the GFAP+ cells were mostly non-multiplying (data not shown), and their morphology differed from that of the RCM-treated astrocytes, being closer to that of prototypic differentiated astrocytes. FGF2 treatment alone did not induce GFAP+ cells (Fig. 7T), but in combination with LIF it yielded proliferating GFAP+ cells with radial glial morphology (Fig. 7V). The addition of VEGF to LIF+FGF2-treated cultures did not affect the yield of these cells (Fig. 7X). In contrast, combined treatment with BMP2 abolished the radial morphology of the GFAP+ cells and turned them into flat cells (Fig. 7W,Y). This is consistent with the fact that BMPs induce prototypic mature astrocytes^45^. Based on these findings, we eliminated BMPs, and tested if the combination of LIF+FGF2+VEGF mimicked the effect of RCM on adult neurogenesis. Infusion of the combined proteins also resulted in an increase in B- and A-type cells in the SVZ (Fig. S7). However, the extent of the increase was smaller than in RCM-infused SVZs, and the areas of DCX+ neuroblasts in the RMS were not significantly increased (Fig. S7G–I), suggesting that the protein combination needs to be further optimized to mimic the effects of RCM.

To further characterize the molecules secreted by Raf-transduced NSCs, we performed a mass spectrometric identification of proteins in the CM (Fig. S8 and Table S2). We detected 77 proteins present in the RCM but not the CCM, and 91 proteins were detected in both RCM and CCM; the levels of 6 of these were >5 fold greater in RCM than in CCM (Fig. S8B and Table S2). The results of an ontology analysis for the RCM-specific and RCM-enriched proteins are shown in Fig. S8C.

## Discussion

Raf-Erk is one of the most common intracellular signals; it confers a variety of NSC behaviors such as NSC self-renewal, and neuronal and astrocytic differentiation. However, no studies have systematically addressed the question of how this signal is associated with multiple, even opposing NSC behaviors. Using ca-Raf transduction, we showed that NSCs in which the Raf-Erk signal is constitutively activated become neurons. Previous studies have also observed neuronal differentiation by cultured NSCs in response to FGF2^22^,^28^,^29^. The neurogenesis promoted in those studies occurred at relatively low concentrations of FGF2 and in NSC cultures plated at low cell densities compared to those in the more common studies demonstrating FGF2-induced NSC proliferation. We showed here that the cell density, but not the Raf-Erk dose, is critical for controlling the choice of proliferation and neuronal differentiation. In fact, we found that neuronal yields were greater at higher levels of Raf-Erk signal activation provided the NSCs were plated at low cell density. In addition to the main Ras-Raf-Erk signal, previous studies showed that FGF2 treatment also activated intracellular CREB signaling via an FGF2-inducible CREB kinase or other pathways, and that CREB activation might be responsible for the neuronal differentiation induced by FGF2 treatment^28^,^29^. Interestingly, along with Erk activation, activated (phosphorylated) CREB (pCREB) levels were also increased in our NSC cultures transduced with ca-Raf (Fig. S2A), probably via ribosomal S6 kinase (RSK) which is downstream in the Raf-Erk pathway^46^. The increased neuronal yields in ca-Raf-transduced cultures were abolished by co-transduction with a dominant negative-CREB (dn-CREB) (Fig. S2B–E). cAMP treatment further increased neuronal differentiation in the ca-Raf-transduced cultures (Fig. S2F–J), and was blocked by the Erk inhibitor PD09825 (Fig. S2K–N). Collectively these observations suggest that pCREB acts downstream in concert with other signaling molecules to induce neuronal differentiation.

On the other hand, Raf-Erk activation in NSCs in response to ca-Raf expression promoted cell proliferation as well as astrocyte marker expression at high cell densities. Consistent with this, a previous study reported that direct deletion of B-Raf or Erk1/2 in the developing CNS resulted in a reduction of proliferating astrocytes and loss of glial properties, while overexpression of these factors had the opposite effects^12^,^13^. We show in this study that an auto/paracrine process underlies Raf-Erk-mediated cell proliferation/astrocyte marker expression.

Raf-Erk is also the main signal activated by FGF2, but FGF2 treatment induces NSC proliferation without astrocyte marker expression in normal NSC cultures. There are two possible mechanisms underlying the different ca-Raf and FGF effects: (1) other signals activated by FGF may block the synthesis of the astrogenic molecules such as LIF/BMPs induced by Raf-Erk signaling, or (2) other signals may make the NSCs non-responsive to astrogenic molecules (for example, by inducing methylation of astrocytic gene promoters)^47^. Interestingly, LIF and BMP4 expression in ca-Raf-transduced NSCs was significantly reduced in the presence of FGF2 (Fig. S9), supporting the first mechanism.

Previous studies have shown that embryonic NSCs are the precursors of RGCs and/or adult-type NSCs^4^,^5^,^34^. It has been reported that Ras-Raf-Erk signaling regulates RGC formation via Ets-1 during Xenopus embryogenesis^48^. Our observation of the Raf-Erk-mediated auto/paracrine role provides further insight into the mechanism of RGC/adult NSC generation. In various types of cells and tissues, the Ras-Raf-Erk pathway provides a positive signal for stem cell proliferation, which is the first and critical event in regeneration (for review, see ref. 49). Sustained Erk activation has been reported to be the key component of the mechanism mediating tissue regeneration in Salamander, a regeneration-competent species^50^. In this study, we showed that infusion of RCM into the SVZ of adult mouse brains promoted a series of CNS regeneration processes, namely expansion of SVZ-NSCs, neuroblast formation and migration to the injured site. Similarly, previous studies^51^,^52^,^53^,^54^ showed that recovery after brain injury was promoted by infusion of FGF, VEGF, or LIF, which we showed to be the major components of RCM. When this combination of cytokines was infused, regenerative processes were facilitated but the effect was not as strong as that obtained with RCM infusion. In order to further identify molecules possibly associated with the RCM effects, we carried out mass spectometric protein identification. The most abundant protein in RCM was tissue inhibitor of metalloproteinase I (TIMP-1) (Table S2), which has previously been reported to play roles in brain development and repair^55^. Interestingly, vimentin, an intermediate filament protein specific for NSC-like and activated astrocytes, was also abundant only in RCM. The secretion of vimentin from activated macrophages and specific roles for the secreted vimentin have been reported^56^. It would be of interest to determine the role of the vimentin released from NSCs upon Raf-Erk activation. An increase in the level of insulin-like growth factor binding protein-2 (IGFBP-2) is responsible for RCM-induced NSC proliferation since it is required for the actions of insulin-like growth factor in NSC self-renewal (for review, see ref. 57). The level of Cystatin-C was also more than 2-fold elevated in RCM. Cystatin-C is an auto/paracrine cofactor required for FGF2-responsive NSC proliferation^58^. Destrin, Moesin, Gelsolin, and Plastin 3 (T-isoform) were also found to be abundant in RCM. Based on their shared functions in regulating the cytoskeleton, they could be involved in changes in cell proliferation, morphology, and neuronal migration observed in RCM-treated cultures and brains. The roles of the identified proteins should be examined in future studies in order to define optimum combinations of cytokines for stimulating brain regeneration.

In conclusion, we have shown that Raf-Erk signal activation in NSCs differentially modulates neuronal differentiation and the acquisition of the cell proliferation/astrocytic phenotype via intrinsic and extrinsic auto/paracrine processes, respectively. These observations were made by selectively activating Raf-Erk in cultured NSCs, mainly by overexpressing ca-Raf, which is a somewhat artificial process. Nevertheless, the value of this study is that it is the first to address the unanswered question of the multiple/opposing effects of Raf-Erk on NSC behaviors, and that it provides an example of how molecular approaches dissecting NSC behaviors can be used in regenerative medicine. In addition, based on the role of Raf-Erk in glioma formation (for reviews, see refs 59, 60, 61, 62, 63, 64, 65), the findings observed in this study may have an important relevance to brain tumor fields.

## Materials and Methods

### Cell cultures

#### NSC cultures from rodent embryonic cortices

NSCs were cultured from embryonic cortical tissue of rats (Sprague-Dawley, SD) at embryonic days 13–14 (E13–14, DBL, Korea) or of mice (C57BL/6, E11–12) expressing eGFP under the control of the human *Gfap* promoter (*pGfap-eGFP*). Briefly, cells dissociated from cortical tissue were plated on culture surfaces pre-coated with poly-L-ornithine (PLO; 15 ug/ml; Sigma-Aldrich, St Louis, MO)/fibronectin (FN; 1 ug/ml; Sigma-Aldrich), and NSCs were grown in N2 medium supplemented with FGF2 (20 ng/ml; R&D Systems, for rat culture) or FGF2 + epidermal growth factor (EGF, 20 ng/ml; R&D Systems, for mouse culture). To obtain a homogenous NSC population, the *in vitro* expanded NSCs were dissociated and re-plated on freshly prepared PLO/FN-coated dishes, and the passaged cultures were used in all our experiments. To generate confluent cell cultures, NSCs were plated at 3–4 × 10^4^ cells/cm^2^ and maintained at >50% of cell confluence. For the cultures with low cell confluence (5–10%), NSCs were plated at 0.5–1 × 10^4^. Differentiation of NSCs was induced by withdrawal of the mitogen(s).

#### Adult SVZ-NSC cultures

NSCs were derived from the SVZ of the lateral ventricle of adult mouse brains (male, 8–10 weeks, C57BL/6). The adult SVZ-NSCs were grown in the form of floating cell aggregates (neurospheres) in FGF2+EGF-supplemented N2 medium. The neurospheres were dissociated into small cell clusters or single cells using Accutase (Stemcell Technologies, Vancouver, BC, Canada), attached to PLO/FN-coated dishes, and induced to differentiate in N2 by withdrawal of the mitogens. In the sphere-forming assay, the spheres (primary) were dissociated into single cells, and seeded at 150 cells per well in 96-well plates. The number and size of spheres (secondary) formed after 5 days were assessed.

#### Astrocyte cultures

Cortical tissues of mouse pups (ICR) on postnatal day 5 were removed and triturated in Dulbecco’s modified Eagle’s medium (DMEM; Life Technologies) containing 10% fetal bovine serum (FBS; HyClone, Logan, UT) and plated in 75-cm^2^ T-flasks. When cell growth reached 80–90% confluence, the glia were harvested with 0.1% trypsin and prepared for use by plating on poly-D-lysine (PDL; Sigma-Aldrich)-coated culture surfaces. Astrocytes were isolated by removing microglia with gentle shaking.

### Virus production and transduction

The cDNA of ca-Raf, which was made to constitutively associate with the plasma membrane by engineering the CAAX motif^26^, was inserted into *pCL* or *pCL-IRES-eGFP* retroviral vectors. Lentiviral vectors (*pCDH*, System Biosciences, Mountain View, CA) were engineered with Nurr1 and *dnCREB* cDNAs. Retroviruses and lentiviruses expressing the transgenes were produced as described previously^66^. Control viruses with empty vectors were also generated for mock-transduced controls. Virus titers were determined using a QuickTiter Retrovirus Quantitation Kit (Cell Biolabs Inc., San Diego, CA) and QuickTiter HIV Lentivirus Quantitation Kit (HIV p24 ELISA)(Cell Biolabs Inc). For viral transduction, NSCs were incubated with viral supernatant for 2–3 hours (retro-virus) or 6 hours (lenti-virus), followed by a medium change.

### Preparation of CM

Four days after transduction, fresh N2 medium (or HBSS) was added to the transduced cultures, and conditioned medium (RCM or CCM) was collected every day for 4 days. Nurr1-CM was similarly prepared from Nurr1-transduced NSC cultures as described previously^37^. The volume of the CMs was adjusted to 1.5 ml/10^6^ transduced cells (0.1–0.13 mg of protein/ml; Bradford assays on 3 independent sets of CCM and RCM after ultrafiltration using Amicon ultra-15 centrifugal filter units (MWCO, 30 kDa; Merck Millipore)), filtered at 0.45 μm, and kept at −80 °C until use. The CMs were used directly in the *in vivo* experiments and mass spectrophotometry/protein identification, or were diluted with N2 medium (1:1, v:v) before adding to cells in cultures.

### Immunofluorescence staining

Cultured cells and cryosectioned brain slices were fixed in freshly prepared 4% paraformaldehyde in PBS, and blocked with 1% (for cultured cells) or 3% (for brain slices) BSA /0.3% Triton X-100 in PBS at room temperature for 1 hour, and then followed by incubation with primary antibody overnight. The following primary antibodies were used: mouse monoclonal antibodies including Ki67 (1:100, Novocastra^TM^ Leica Biosystems); TUJ1 (1:500, Covance, Princeton, NJ); phospho-ERK1/2 (pERK1/2; 1:1000, Cell Signaling Technology, Beverly, MA); eGFP (1:400, Roche. Indianapolis, IN); glial fibrillary acidic protein (GFAP; 1:1000, MP Biomedicals, Santa Ana, CA); Nestin (1:1000, BD Biosciences, Frankin Lakes, NJ); Vimentin (1:100, Merck Millipore); NeuN (1:200, EMD millipore, Billerica, ma); Microtubule-associated protein 2 (MAP2; 1:200, Sigma); BrdU (1:200, AbD Serotec Ltd., Oxfordshire, UK); MASH1 (1:500, BD Bioscience); epidermal growth factor receptor (EGFR, 1:500, Abcam, Cambridge, MA) and rabbit polyclonal antibodies including SOX2 (1:200, Merck Millipore, Billerica, MA); eGFP (1:2000, Invitrogen^TM^ Life Technologies); GFAP (1:400, DAKO, Carpinteria, CA); TUJ1 (1:1000, Covance); pHH3 (1:500, Invitrogen^TM^ Life Technologies) and goat polyclonal doublecortin (DCX, 1:500, Santa Cruz Biotechnologies). To visualize the antibodies, secondary antibodies tagged with Cy3 or Cy5 (Jackson ImmunoResearch Laboratories, West Grove, PA) or Alexa Fluor 488 (Invitrogen^TM^ Life Technologies) were used. Stained images were captured using an epifluorescence microscope (DM5000B, Leica Micro-systems GmbH, Wetzlar, Germany) or a confocal microscope (TSC SP5, Leica) in Hanyang Research Facilities Center at Hanyang University (Seoul, Korea). Immunoreactive cells in the SVZ, RMS, and regions migrating to the TBI were counted in every 6 sections, and the total numbers of these cells in those regions were obtained by multiplying by 6. Two or three sections anterior and posterior to the needle site were excluded from the cell counting. In case of the migrating eGFP+/Dcx+ cell counting, all sections containing immunoreactive cells in the migrating regions (3–4 sections/animal) were counted.

### Real-time PCR analyses

Total cellular RNA was prepared using Tri Reagent (Molecular Research Center) according to the manufacturer’s instructions. cDNA synthesis was performed using Superscript kit (Invitrogen). Real-time PCR was performed on a CFX96 Real time system using iQ SYBR green supermix (Bio-Rad). All gene expression values were normalized to those of glyceraldehyde 3-phosphate dehydrogenase. PCR primers are summarized in Table S1.

### Western blot analysis

Whole cell lysates were extracted in lysis buffer, subjected to denaturing sodium dodecyl sulfate (SDS) polyacrylamide gel electrophoresis, and transferred to a nitrocellulose membrane. Transferred proteins were blocked in 3–5% bovine albumin serum (BSA, Sigma) in Tris-buffered saline with 0.1% Tween 20. The blots were incubated with anti-mouse pERK1/2 (1:1000), ERK (1:1000), TUJ1 (1:1000, Covance), MAP2 (1:1000, Sigma-Aldrich) and anti-rabbit c-Raf (1:1000), CREB (1:1000), pCREB (1:1000, all from Cell Signaling Technology) antibodies followed by IgG antibodies conjugated with peroxidase (1:2000, Cell Signaling Technology). Bands were visualized by enhanced chemiluminescence (ECL; Thermo Scientific, Rockford, IL).

### FACS analysis

Cells were harvested, fixed in cold methanol, and labeled with 50 μg/ml propidium iodide (PI; Invitrogen^TM^ Life Technologies) containing 125 U/ml protease-free RNase (Roche). Harvested cells were filtered through a 95-um pore size nylon mesh. 10,000 stained single cells were activated with a 488 nm laser, measured through a 585/42 nm band pass filter, and analyzed by FACS Canto II (BD Biosciences). The FACS analyses were performed by the Hanyang Research Facilities Center.

### Glutamate uptake

Cells were washed twice in Tissue Buffer (5 mM Tris, 320 mM sucrose, pH7.4) and exposed to 10 uM glutamate in either Na+-containing Krebs buffer (120 mM NaCl, 25 mM NaHCO_3_, 5 mM KCl, 2 mM CaCl_2_, 1 mM KH_2_PO_4_, 1 mM MgSO_4_, 10% glucose) or Na+-free Krebs (120 mM choline-Cl and 25 mM Tris-HCl) for 10 min at 37 °C. Uptake was stopped by placing the cells on ice and washing them twice with Wash Buffer (5 mM Tris/160 mM NaCl pH7.4). Cells were collected and homogenized in 100 ul of assay buffer and glutamate amounts in the cell homogenates were measured using a glutamate assay kit (Abcam, ab83389). Na+-dependent uptake was determined by subtracting Na+-free counts from total counts in the presence of Na+.

### Osmotic pump implantation and histological procedure

Mice (Male, C57BL/6, 50–100 g) were used for the *in vivo* experiments. All procedures for animal experiments were approved by the Institutional Animal Care and Use Committee (IACUC) at Hanyang College of Medicine under the approval number 2013-0153A. Animals were housed in a specific pathogen-free barrier facility with a 12-h light/dark cycle and maintained on standard chow (5053PicoLabR Rodent Diet 20). Experiments were performed in accordance with the NIH guidelines. Cryogenic TBI was imposed by placing an iron rod (diameter: 5 mm) pre-chilled in liquid nitrogen on the cranium (2 cm rostral, 0.3 cm right to the bregma) for 30–60 seconds. Under anesthesia (by i.p. injection using a mixture of zoletil (0.01 mg/kg) and rompun (0.2 mg/kg)), an osmotic pump (ALZET pumps 1007D, ALZET Osmotic Pumps, Cupertino, CA) filled with CSF (control), CCM, RCM, or a mixture of cytokines (LIF+FGF2+VEGF) was implanted subcutaneously in the back. A stainless steel brain cannula (ALZET brain infusion kit 2) connected to the pump was inserted into the right lateral ventricle (AP: −0.5; ML: −1.2; DV: −2.5) with respect to the bregma/midline intersection, and fixed with adhesive gel (Loctite 454, ALZET Osmotic Pumps). The mice were killed 6 days after surgery. Proliferating cells were labeled by injecting BrdU (50 mg/kg) i.p for 1 hr before mice were killed. In certain experiments, retroviruses expressing eGFP (10^11^TU) were locally injected to the right SVZ tissue (AP: +0.02; ML: −0.14; DV: −0.20) 2 days prior to the TBI, and the eGFP-expressing cells were chased. Brains were perfused with 4% paraformaldehyde, immersed in 30% sucrose in PBS overnight, and coronally sectioned on a freezing microtome (Leica Bio-systems, Newcastle, U.K). Free-floating brain sections (40 μm thick) were subjected to immunohistochemistry. Unbiased stereology (TissueFAXs, TissueGnostics GmbH, Vienna, Austria) was applied to the immunoreactive cell counting in the brain.

### Mass spectrometric protein identification

CMs were concentrated using 3 kDa cut-off MWCO membranes (Merck Millipore) at 4000 rpm. The resulting concentrates were collected and samples containing 100 ug of protein were used in each LC-MS/MS analysis. In-gel trypsin digestion and LC-MS/MS analysis using a LTQ Orbitrap mass spectrometer (Thermo Scientific) equipped with NSI sources (San Jose, CA) were performed as previously described^67^. For database searching, tandem mass spectra were extracted by a Thermo Proteome Discoverer 1.3.0.339. Charge state deconvolution and deisotoping were not performed. All MS/MS samples were analyzed using Sequest (XCorr Only) (Thermo Fisher Scientific, San Jose, CA, USA; version 1.3.0.339) and X! Tandem (The GPM, thegpm.org; version CYCLONE (2010.12.01.1)). Sequest (XCorr Only) was set up to search RAT 150107.fasta (29,376 entries) assuming the digestion enzyme trypsin. X! Tandem was set up to search a subset of the RAT 150107 database also assuming trypsin digestion. Sequest (XCorr Only) and X! Tandem were searched with a fragment ion mass tolerance of 0.80 Da and a parent ion tolerance of 20 PPM. Scaffold (version Scaffold_4.4.1, Proteome Software Inc., Portland, OR) was used to validate MS/MS based peptide and protein identification. Peptide identifications were accepted if they were established at greater than 90% probability by the Scaffold Local FDR algorithm, and protein identifications were accepted if they were established at greater than 95% probability and contained at least 2 identified peptides. Protein probabilities were assigned by the Protein Prophet algorithm^68^. Proteins that contained similar peptides and could not be differentiated based on MS/MS analysis alone were grouped to satisfy parsimony. Proteins were annotated with GO terms from NCBI (downloaded Feb 5, 2015)^69^.

### *Gfap* promoter luciferase assay

The *Gfap* promoter [wild-type (*GF1L*) and *Gfap* promoter mutant in which the STAT3 binding sequence is deleted (*GFlL-SBSPM*)^70^ (kindly provided by Dr. Kinichi Nakashima from Kyushu University) were cloned into a *pGL3* luciferase plasmid (Promega). The plasmids were co-transfected into cells with a *β-galactosidase* expression vector as a reference using Lipofectamine 2000 (Invitrogen). Two days after transfection, cell lysates were collected and subjected to luciferase assay (BD Bioscience). Luciferase activity was measured and normalized to β-galactosidase activity.

### Cell counting and statistical analysis

Immunoreactive or DAPI-stained cells were counted in at least 20 random areas of each culture coverslip using an eyepiece grid at a magnification of 200 or 400×. Statistical comparisons between two groups were made by Student’s t test. One-way ANOVA followed by Turkey’s HSD *post hoc* analysis (SPSS^®^ Statistics 21; IBM Inc. Chicago, IL) was used when more than two groups were compared. Data are expressed as mean ± SEM. The n, p-values, and statistical analysis methods are indicated in the figure legends.

## Additional Information

**How to cite this article**: Rhee, Y.-H. *et al*. Neural stem cells secrete factors facilitating brain regeneration upon constitutive Raf-Erk activation. *Sci. Rep.* **6**, 32025; doi: 10.1038/srep32025 (2016).

## Supplementary Material

Supplementary Information
