# Supplementary material for: Neural stem cells secrete factors facilitating brain regeneration upon constitutive Raf-Erk activation
Source: Sci Rep. 2016 Aug 24;6:32025. doi: 10.1038/srep32025 (PMC4995508; doi:10.1038/srep32025)
Supplement: Supplementary Information [file srep32025-s1.pdf]

Supplementary data

Neural stem cells secrete factors facilitating brain regeneration upon constitutive Raf-Erk activation

(Running title: Therapeutic use of Raf-Erk activation in NSCs)

Yong-Hee Rhee[1,2,8], Sang-Hoon Yi[1,2,8], Joo Yeon Kim[3], Mi-Yoon Chang[1,2], A-Young Jo[1,2], Jinyoung Kim[1,2], Chang-Hwan Park[2,5], Je-Yoel Cho[6], Young-Jin Choi[7], Woong Sun[3], Sang-Hun Lee[1,2,5,§]

[1]Department of Biochemistry and Molecular Biology, College of Medicine, Hanyang University, Seoul, Korea

[2]Hanyang Biomedical Research Institute, Hanyang University, Seoul, Korea

[3]Department of Anatomy, College of Medicine, Korea University, Seoul, Korea

[4]Department of Microbiology, College of Medicine, Hanyang University, Seoul, Korea

[5]Graduate School of Biomedical Science and Engineering, Hanyang University, Seoul, Korea

[6]Department of Biochemistry, BK21 PLUS Program for Creative Veterinary Science Research and Research Institute for Veterinary Science, College of Veterinary Medicine, Seoul National University, Seoul, South Korea

[7]ProtAnBio, Co., Seoul, South Korea

[8]Co-first author

§Author for correspondence

Department of Biochemistry and Molecular Biology

College of Medicine, Hanyang University

Seoul, KOREA

Phone: 82-2-2220-0625

Email: leesh@hanyang.ac.kr

Supplementary Figures and legends

Figure S1

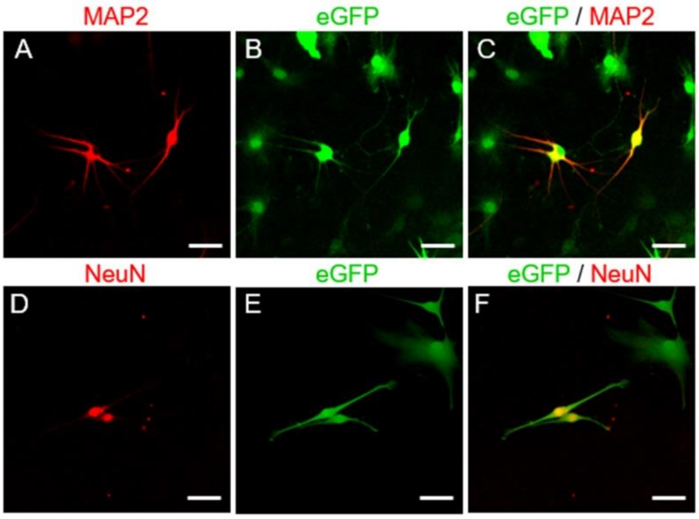

Fig. S1. Expression of the mature neuron-specific markers MAP2 (A-C), NeuN (D-F) in Raf-transduced eGFP+ cells. NSCs derived from rat embryonic cortices were plated at 5 $x10^3$ cells $/cm^2$ and transduced with bicistronic *ca-Raf-IRES-eGFP* ($2x10^{10}$TU). The low density cultures were differentiated for 10 days and then immunostained against eGFP/MAP2 and eGFP/NeuN. Scale bar, 30μm.

Figure S2

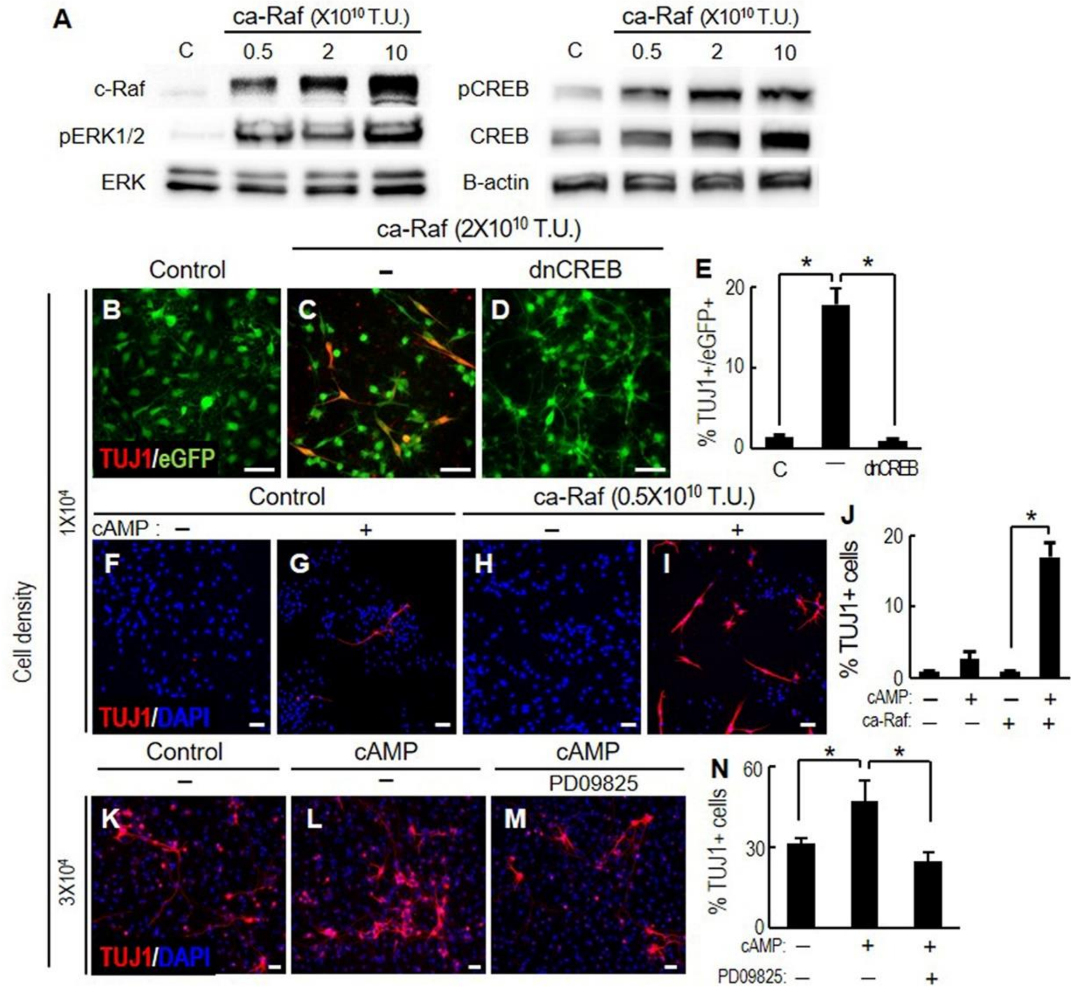

Fig. S2. CREB mediates neuronal differentiation induced by Raf-Erk activation. E14 cortical NSCs were plated at 1x (B-J) or $3x10^4/cm^2$ (A and K-N), and transduced (or not) the following day. Analyses were carried out 4-6 days after transduction. A, CREB activation estimated from phosphorylated CREB (pCREB) protein levels obtained by WB analysis. B-E, CREB inhibition abolishes neuronal differentiation induced by ca-Raf expression. TUJ1+ neuronal yields were estimated in cultures transduced with empty control (B), ca-Raf (C), and ca-Raf+ dominant negative CREB (dn-CREB) (D). The transduced cells were labeled with *eGFP* using the bicistronic *–IRES-eGFP* construct. F-J, cAMP treatment enhances ca-Raf-induced neuronal yields. K-N, cAMP-induced neuron formation is abolished by the Erk inhibitor PD98059 (10μM).*p<0.005, n=3-5 cultures for each group, one-way ANOVA. Scale bars, 50μm.

Figure S3

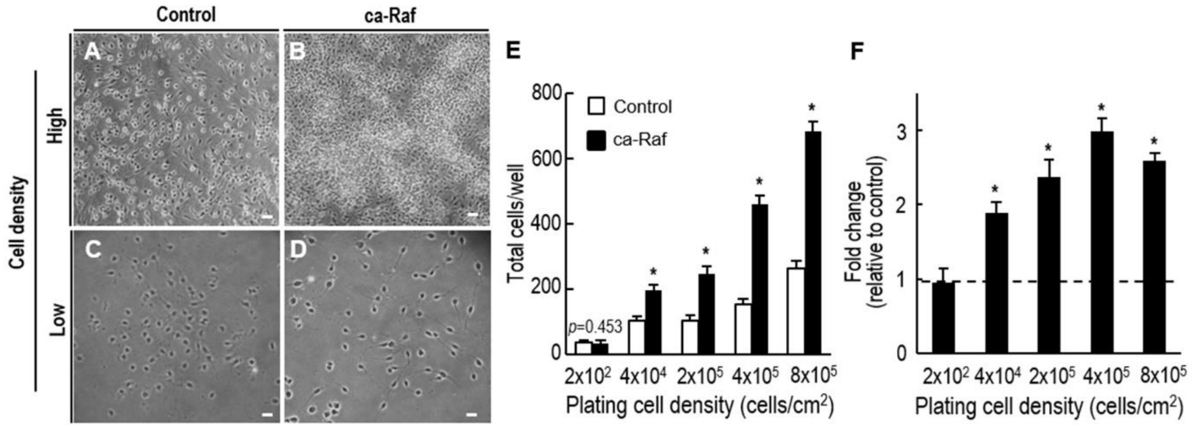

Fig. S3. Cell density-dependent effect of Raf-Erk activation on cell proliferation. Cortical NSCs were plated at the cell densities indicated, and transduced with ca-Raf or mock control the following day. The cultures were fixed and total viable cells were counted 4 days after transduction. A-D, Representative phase contrast images 4 days after cell plating at 4×10^5 cells/cm^2 (high) and 2×10^2 cells/cm^2 (low). E and F, The data are total cells/well (E) and % cell numbers relative to the respective controls (F) at each cell density. *p<0.01, n=3 culture wells for each datum, one-way ANOVA. Scale bars, 50μm.

Figure S4

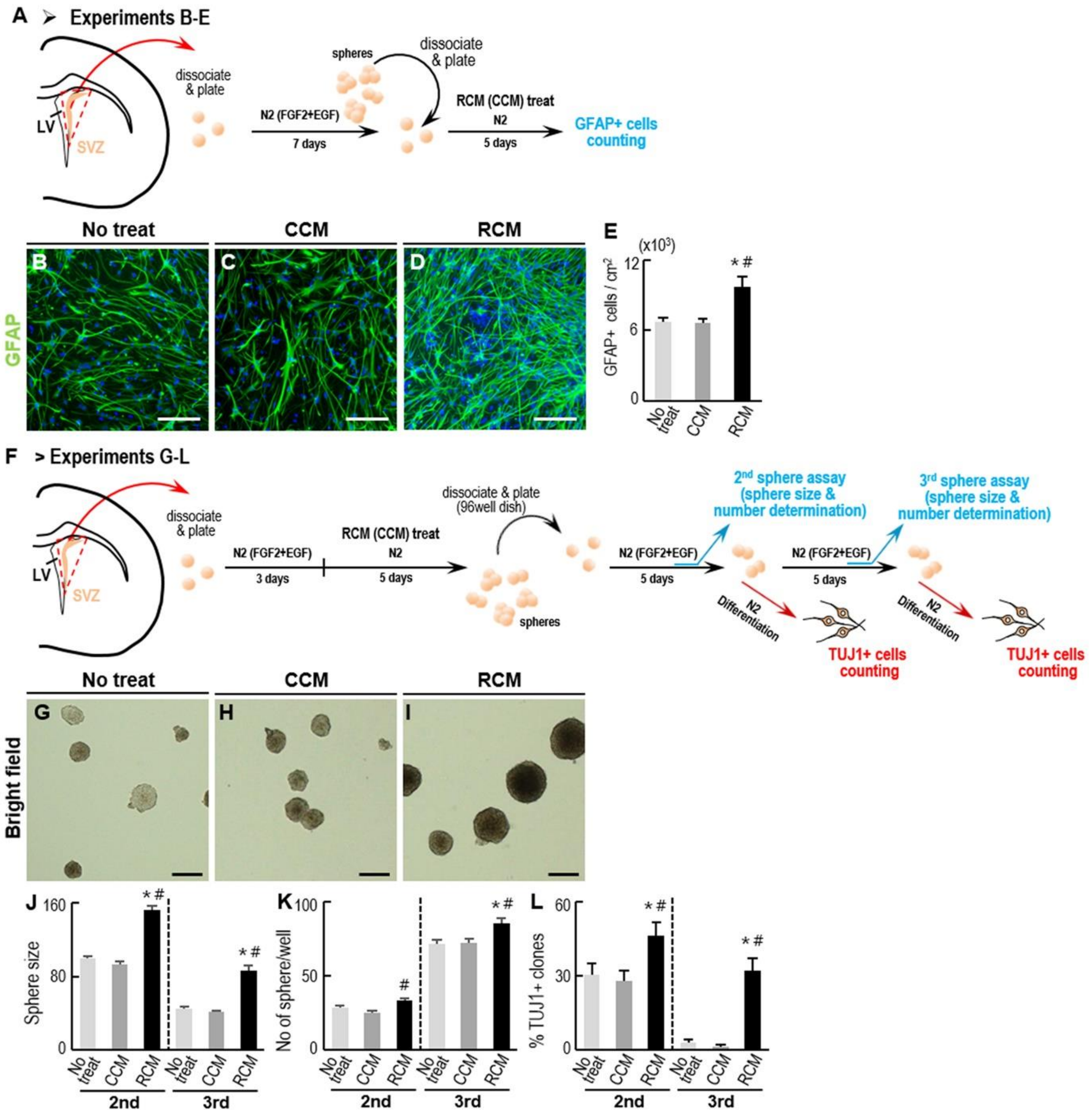

Fig. S4. The effect of RCM on adult brain-derived NSCs. A-E, RCM effect on GFAP+ adult NSC number. A, schematic of the experimental procedure in B-E. Cells dissociated from the SVZs were cultured in N2 supplemented with FGF2+EGF (20 ng/ml each) for 7 days. The spheres formed were dissociated, plated on PLO/FN-coated dishes, and cultured in N2 medium in the presence of CM or not. Five days later, GFAP+ cells were estimated (B-E). Significantly different from the untreated* and from the CCM-treated# at p<0.001, n=3 culture wells. F-L, Neurosphere-forming assays. Schematic of the experimental procedure is shown in F. SVZ-derived neurospheres were treated with CM for 5 days, then dissociated into single cells, and plated at 150 cells/well in 96-well plates. The

sizes and numbers of spheres (secondary) formed after 5 days were assessed (G-J). The secondary spheres were collected and subjected to tertiary sphere forming assays in the same way. The secondary and tertiary spheres were attached to PLO/FN-coated dishes and induced to differentiate in N2 medium. % TUJ1+ colonies were counted after 7 days (L). 120 spheres (J), 40 wells (K), 6 dishes (L) were analyzed. Significantly different from the untreated* and from the CCM-treated# at $p<0.001$, one-way ANOVA. Scale bar, 100μm.

Figure S5

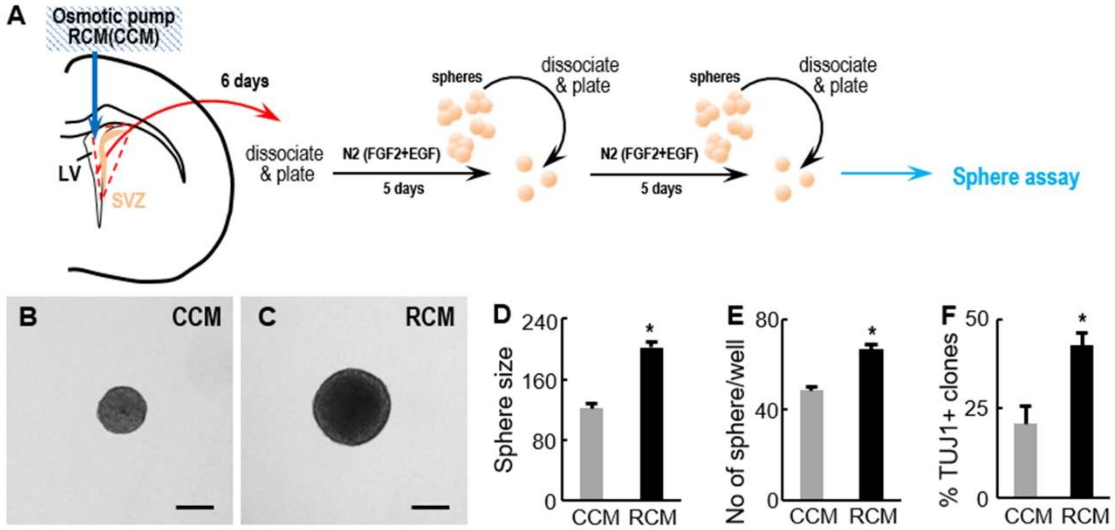

Fig. S5. Sphere forming assays using RCM (or CCM)-infused SVZs. A, RCM (or CCM) were infused into the right LV of adult mice (without TBI) as described in Fig. 5A. Six days after infusion, the ipsilateral SVZs were dissociated and cultured in FGF2 + EGF-supplemented N2 for 5 days. Spheres formed were dissociated into single cells and neurosphere-forming assays were carried out as described in Supplementary Fig. S4. B-F, Sizes (D) and numbers (E) of the spheres were assessed 5 days after plating on 96-well plates. Shown in B and C are representative spheres derived from CCM- and RCM-infused SVZ tissues, respectively. The spheres were plated on PLO/FN-coated dishes and induced to differentiate in N2 medium. TUJ1+ neuron-containing clones were counted 7 days after differentiation (F). 40 spheres (D), 30-40 wells (E), and 200-300 clones (F) were analyzed per animal, and the data are expressed as mean ± SEM of 3 mice. *Significantly different from CCM-infused mice at p<0.001, Student's t-test, n=3 mice for each RCM- and CCM-infused group. Scale bar, 200μm.

Figure S6

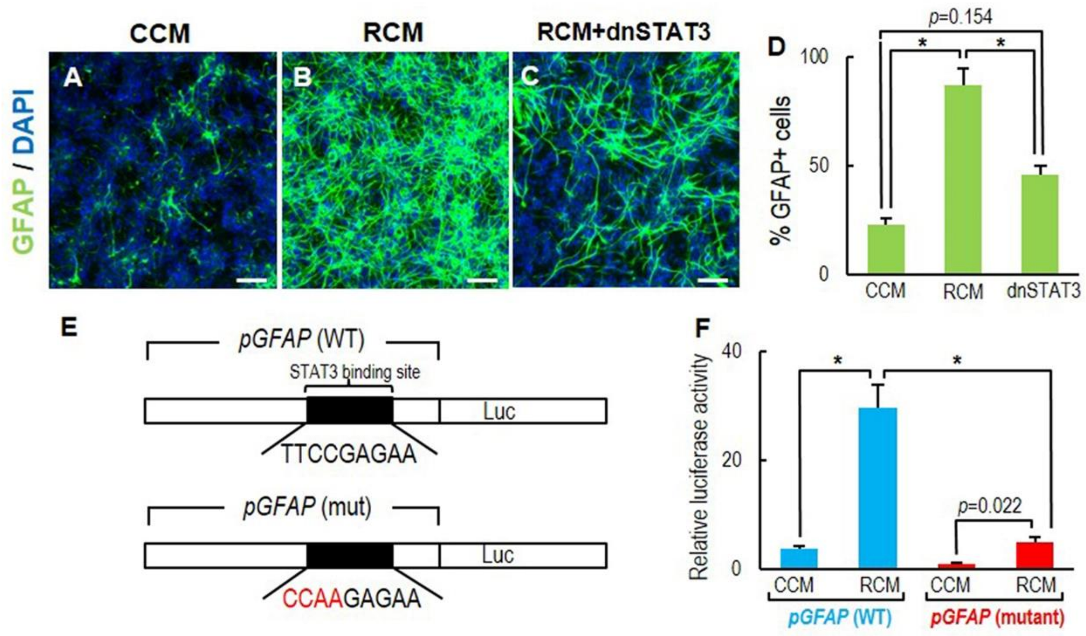

Fig. S6. Jak-STAT signal activation is responsible for astrocytic differentiation induced by RCM treatment. A-D, Enhanced astrocyte yields in RCM-treated cultures are abolished by STAT signal inhibition using a dominant negative STAT3 (dn-STAT3). NSCs cultured from cortices of rat embryos (at E14) were transduced with retroviruses expressing dn-STAT3 (C) or mock vector (A, B). Two days after transduction, the NSCs were treated with CCM or RCM for 4 days. GFAP+ cells were counted 4 days after CM treatment (D). *$p<0.001$, n=3 cultures, one way-ANOVA. scale bar, 50μm. E and F, *GFAP* promoter assay. Schematic drawing of the STAT binding sequences in the luciferase reporters driven by the wild-type (WT) and mutant (mut) *GFAP* promoters (*pGFAP*) (E). The cortical NSCs were transfected with the reporter vectors and luciferase activities were determined in (F). *$p<0.001$, n=4 replicates, one way-ANOVA.

Figure S7

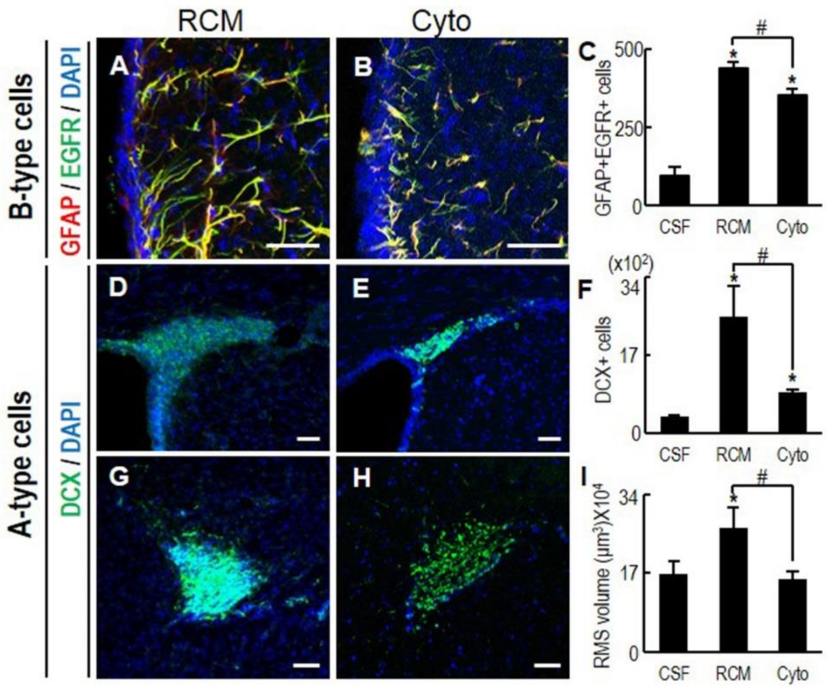

Fig. S7. Effect of the combined cytokines LIF+FGF2+VEGF on in vivo neurogenesis by SVZ-NSCs. The levels of the cytokines LIF, FGF2, and VEGF were determined as described in Fig.7P and they were infused as described in Fig.5A. Cell populations of activated B-type cells (GFAP/EGFR- double positive, A-C) in the SVZ and A cells (DCX+) in the SVZ (D-F) and RMS (G-I) of the cytokine-infused (Cyto) brains were comapared with those of the mice infused with CSF (control) and RCM. Significantly different from the CSF-infused control* and from the RCM-infused# at *p*<0.005, one-way ANOVA, n= 4 (CSF), 7 (RCM), and 4 (Cyto). Scale bar, 50µm.

Figure S8

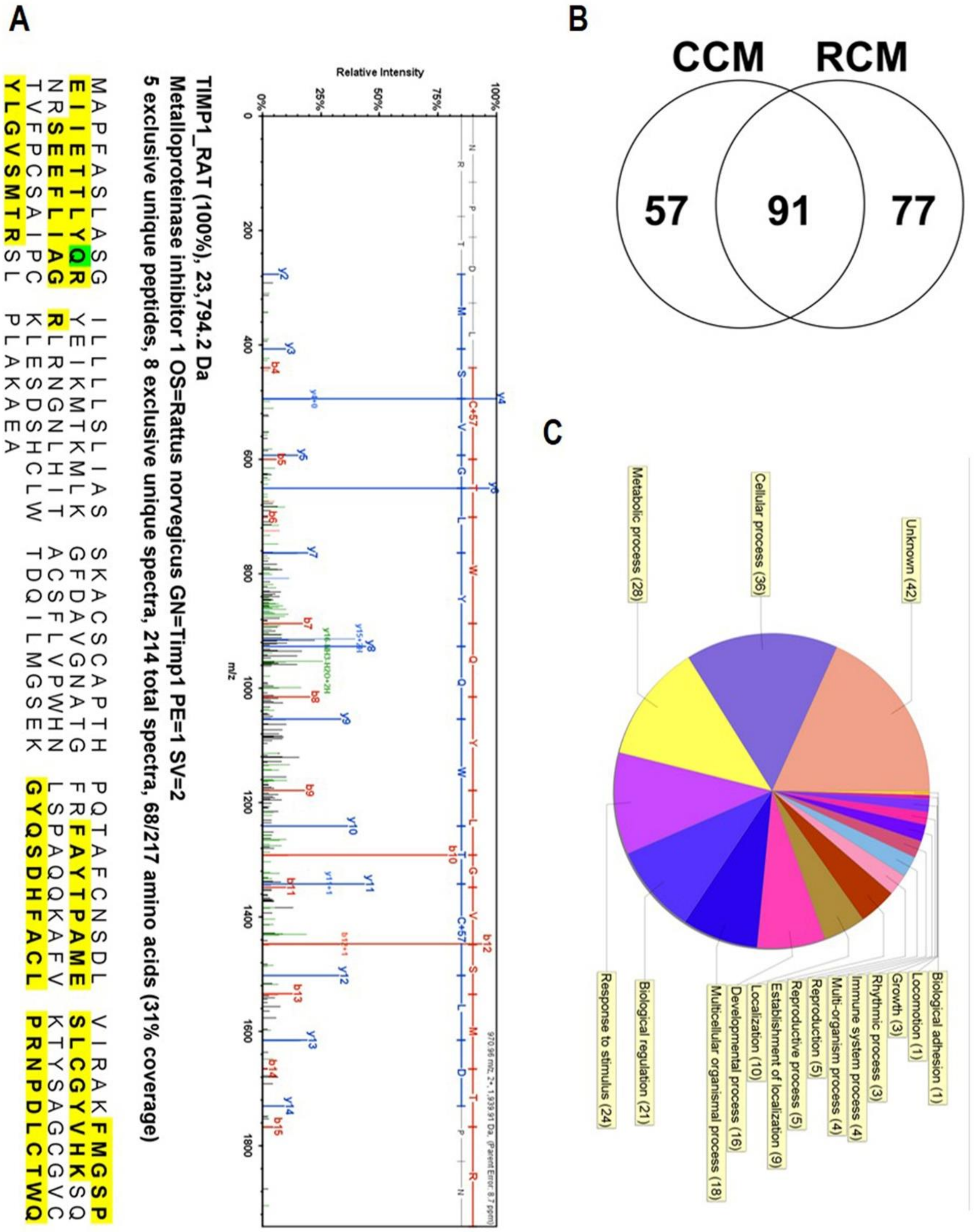

Fig. S8. Identification of proteins in CM by LC-MS/MS. Proteins (100μg) in RCM and CCM were separated by 12% SDS-PAGE and stained with Coomassie Brilliant Blue. The stained proteins were cut into 20 pieces for in-gel trypsin digestion and analyzed. A, Representative LC/MS spectrum of the metalloproteinase inhibitor 1 (TIMP1) identified in RCM. B, Band-diagram exhibiting numbers of the

proteins detected in RCM and CCM. A total 225 proteins were identified, of which 57 and 77 proteins were detected only in the CCM and RCM, respectively, and 91 proteins were detected in both the CCM and RCM. C, Ontologic analysis of the 77 proteins present only in RCM and 6 proteins that were >5 fold higher in RCM than CCM (listed in Table S2). The proteins are classified according to the gene ontology term: "Biological process".

Figure S9

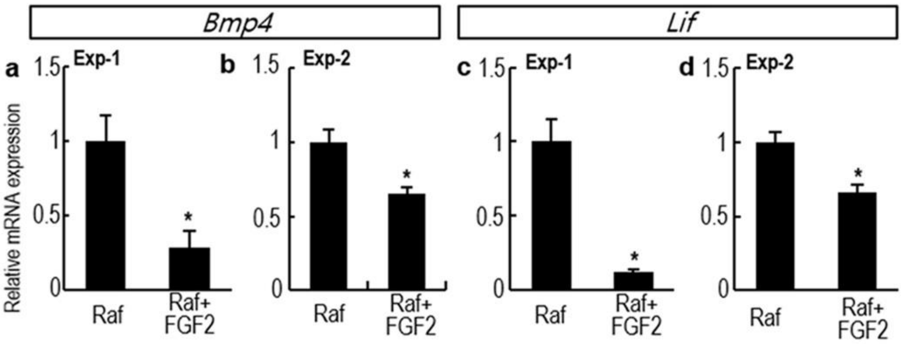

Fig. S9. Real-time PCR analysis of *Bmp4* and *Lif* mRNA expression in ca-Raf-trasnduced NSC cultures in the presence or absence of FGF2 (10 ng/ml). *Significant at $p<0.01$, $n=3$ PCR reactions for each datum, Student's t-test.

Table. S1. Primers for PCR analyses, Related to Figure 4.

Gene symbol	Sequence
Aqp4	F: TTG CTT TGG ACT CAG CAT TG
	R: GGG AGG TGT GAC CAG GTA GA
Bmp2	F: TGA GGC TGC TCA CCA TGT TTG
	R: GTG ACA TCA AAG CTC TCC CAC T
Bmp4	F: ATT GGC TCC CAA CTT CTT CCT TT
	R: CGT GAT GGA AAC TCC TCA CAG T
Bmp7	F: CGC TCC AAG ACT CCA AAG AAC C
	R: CGG TGT CTG GGT TGATGA AGT G
Egfr	F: ACC GTG GAG AGA ATC CCT TT
	R: TTG TTG CTA AAT CGC ACA GC
Fabp7	F: CCA GCT GGG AGA AGA GTT TG
	R: TAA CAG CGA ACA GCA ACG AC
Fgf1	F: CGC AGA CAC CAA ATG AAG AA
	R: TTT CTG GCC GTA GTG AGT CC
Fgf2	F: GAA CCG GTA CCT GGC TAT GA
	R: CCG TTT TGG ATC CGA GTT TA
Gapdh	F: GGC ATT GCT CTC ATT GAC AA
	R: AGG GCC TCT CTC TTG CTC TC
Gfap	F: GCA GAC CTC ACA GAC GTT GCT
	R: AGG CTG GTT TCT CGG ATC TGG
Slc1a3	F: GGA TGG AAA GA TTC CAG CAA
	R: ACC TCC CGG TAG CTC ATT TT
Glt1	F: CCA AAA GCA ACG GAG AAG AG
	R: ACC TCC CGG TAG CTC ATT TT
Lif	F: GGC AAC CTC ATG AAC CAG AT
	R: ACC ATC CGA TAC AGC TCG AC
Nestin	F: GGA GTG TCG CTT AGA GGT
	R: TCC AGA AAG CCA AGA GAA
Notch	F: GGT GCG AGC GCA GTG AAG GA
	R: CCC GCT GCT GCC CTC TTT CC
Sox2	F: TCA CAA CAA TCG CGG CGG CCC
	R: GCG CGG AGA TCT GGC GGA GAA T
Pax6	F: TGT CCA ACG GAT GTG TGA GT
	R: TTT CCC AAG CAA AGA TGG AC
Vegf	F: GTG CAC TGG ACC CTG GCT TTA CT
	R: CGC CTT GCA ACG CGA A
Vimentin	F: AGA TCG ATG TGG ACG TTT CC
	R: CAC ACT GTC TCC GGT ATT CGT

Table. S2. Proteins abundantly detected in the RCM, Related to Figure S8.

#	Identified Proteins	Accession Number	Fold change by sample	Normalized Spectral counts	
				CCM	RCM
**1**	Vimentin OS	G3V8C3_RAT (+1)	RCM only		125
**2**	Metalloproteinase inhibitor 1	TIMP1_RAT	RCM only		160
**3**	Alpha-2-macroglobulin	A2MG_RAT	RCM only		114
**4**	Moesin	A0A096MK30_RAT(+1)	RCM only		51
**5**	Peptidyl-prolyl cis-trans isomerase	Q6AYQ9_RAT	RCM only		93
**6**	Sulfated glycoprotein 1	F7EPE0_RAT (+1)	RCM only		49
**7**	Mannan-binding lectin serine protease 1	MASP1_RAT	RCM only		56
**8**	Carbonic anhydrase 2	CAH2_RAT	RCM only		65
**9**	Uncharacterized protein	D3ZJE2_RAT (+1)	RCM only		57
**10**	Gelsolin	GELS_RAT	RCM only		37
**11**	Myosin-6	MYH6_RAT	RCM only		1
**12**	Histone H3	D3ZJ08_RAT (+6)	RCM only		13
**13**	Uncharacterized protein (Fragment)	F1M9P7_RAT (+1)	RCM only		43
**14**	Chloride intracellular channel protein 1	CLIC1_RAT	RCM only		47
**15**	Heat shock protein HSP 90-alpha	HS90A_RAT	RCM only		43
**16**	Nucleolin	NUCL_RAT (+1)	RCM only		19
**17**	Latexin	LXN_RAT	RCM only		16
**18**	Elongation factor 1-gamma	EF1G_RAT	RCM only		13
**19**	Heterogeneous nuclear ribonucleoprotein K	HNRPK_RAT (+2)	RCM only		14
**20**	Coactosin-like protein	COTL1_RAT	RCM only		6
**21**	Insulin-like growth factor-binding protein 3	IBP3_RAT	RCM only		21
**22**	Destrin	DEST_RAT	RCM only		78
**23**	Amyloid beta A4 protein	A4_RAT	RCM only		6
**24**	Eukaryotic translation initiation factor 5A2 (Predicted)	G3V7J7_RAT (+1)	RCM only		4
**25**	Elongation factor 1-alpha 1	EF1A1_RAT	RCM only		17
**26**	Glutathione S-transferase alpha-4	GSTA4_RAT	RCM only		5
**27**	Proteasome subunit beta type	G3V8U9_RAT (+1)	RCM only		10
**28**	Proteasome subunit beta type-2	PSB2_RAT	RCM only		2
**29**	60S acidic ribosomal protein P0	RLA0_RAT	RCM only		25
**30**	Ubiquitin-conjugating enzyme E2 N	UBE2N_RAT	RCM only		12
**31**	Poly(RC) binding protein 2	Q6AYU5_RAT	RCM only		25
**32**	Pyruvate kinase PKM	KPYM_RAT	RCM only		18
**33**	Protein LOC100362142	D3ZFY8_RAT	RCM only		25
**34**	Protein Snrpd3	M0R907_RAT	RCM only		4
**35**	Prothymosin alpha	PTMA_RAT	RCM only		19
**36**	Actin related protein 2/3 complex, subunit 4 (Predicted), isoform CRA_a	B2RZ72_RAT	RCM only		13
**37**	Carbonyl reductase [NADPH] 1	CBR1_RAT (+1)	RCM only		7
**38**	Thioredoxin (Fragment)	R4GNK3_RAT (+1)	RCM only		9

**39**	14-3-3 protein eta	1433F_RAT	RCM only		90
**40**	Adenylate kinase 2	KAD2_RAT	RCM only		13
**41**	Heterogeneous nuclear ribonucleoprotein F	HNRPF_RAT	RCM only		16
**42**	Prostaglandin E synthase 3 (Fragment)	R9PXR7_RAT (+1)	RCM only		5
**43**	Protein Eea1 (Fragment)	F1LUA1_RAT	RCM only		1
**44**	Protein LOC100910754	D4A0F5_RAT (+2)	RCM only		2
**45**	Plastin 3 (T-isoform), isoform CRA_a	F1LPK7_RAT (+1)	RCM only		12
**46**	Protein Hectd4 (Fragment)	F1LZX5_RAT	RCM only		4
**47**	NudC domain-containing protein 2	NUDC2_RAT (+1)	RCM only		7
**48**	Protein Dag1	F1M8K0_RAT	RCM only		4
**49**	Protein Tln1	G3V852_RAT	RCM only		1
**50**	RCG50226, isoform CRA_a	G3V8P4_RAT	RCM only		4
**51**	Actin-related protein 2/3 complex subunit 3	B2GV73_RAT	RCM only		7
**52**	6-phosphogluconolactonase	6PGL_RAT (+1)	RCM only		8
**53**	Proteasome subunit beta type	F1LNN1_RAT (+1)	RCM only		2
**54**	Clathrin heavy chain 1	CLH1_RAT (+1)	RCM only		1
**55**	Cullin-associated NEDD8-dissociated protein 1	CAND1_RAT	RCM only		4
**56**	Keratin, type II cytoskeletal 1	K2C1_RAT	RCM only		7
**57**	Protein Sh3bgrl3	B2RZ27_RAT	RCM only		2
**58**	Protein Ppp2r1a	Q5XI34_RAT	RCM only		1
**59**	Heterogeneous nuclear ribonucleoprotein C	G3V9R8_RAT	RCM only		10
**60**	Alpha-actinin-4	ACTN4_RAT	RCM only		2
**61**	Peptidyl-prolyl cis-trans isomerase D	PPID_RAT	RCM only		8
**62**	UMP-CMP kinase	KCY_RAT	RCM only		1
**63**	Uncharacterized protein (Fragment)	F1LYE1_RAT	RCM only		1
**64**	72 kDa type IV collagenase	E9PSM5_RAT (+1)	RCM only		7
**65**	Protein LOC102547754	D4ADL2_RAT	RCM only		2
**66**	Thimet oligopeptidase	THOP1_RAT	RCM only		2
**67**	Protein LOC100360057 (Fragment)	F7FLF2_RAT (+1)	RCM only		4
**68**	Importin subunit beta-1	F2Z3Q8_RAT (+1)	RCM only		4
**69**	Nidogen-1	F1LM84_RAT	RCM only		2
**70**	Clusterin	CLUS_RAT (+1)	RCM only		4
**71**	Serine/threonine-protein phosphatase PP1-alpha catalytic subunit	PP1A_RAT	RCM only		2
**72**	Protein Chd4	E9PU01_RAT	RCM only		1
**73**	Heterogeneous nuclear ribonucleoprotein D, isoform CRA_b	G3V6A4_RAT (+3)	RCM only		2
**74**	Glutathione S-transferase omega-1	GSTO1_RAT (+1)	RCM only		2
**75**	Ubiquitin-conjugating enzyme E2 variant 2	UB2V2_RAT	RCM only		3
**76**	Protein Sorcs1	F1LUZ4_RAT	RCM only		1

**77**	Protein LOC100362339	D4A6G6_RAT (+1)	RCM only		1
**78**	Carboxypeptidase E	CBPE_RAT	9.1	20	179
**79**	Glia-derived nexin	G3V7Z4_RAT	8.6	55	469
**80**	SPARC-like 1 (Mast9, hevin), isoform CRA_a	G3V7X5_RAT (+1)	7.2	64	457
**81**	Transketolase	G3V826_RAT (+1)	6.9	9	63
**82**	Insulin-like growth factor-binding protein 2	IBP2_RAT	6.5	41	264
**83**	Heat shock cognate 71 kDa protein	HSP7C_RAT	6.4	21	136
